# Stochastic dynamic causal modelling of fMRI data: Should we care about neural noise?

**DOI:** 10.1016/j.neuroimage.2012.04.061

**Published:** 2012-08-01

**Authors:** J. Daunizeau, K.E. Stephan, K.J. Friston

**Affiliations:** a Wellcome Trust Centre for Neuroimaging, University College of London, UK; b Laboratory for Social and Neural Systems Research, Dept. of Economics, University of Zurich, Switzerland; c Brain and Spine Institute, Paris, France; d Translational Neuromodeling Unit (TNU), Institute of Biomedical Engineering, University of Zurich & ETH Zurich, Switzerland

**Keywords:** DCM, Network, System identification, Neural noise, Nonlinear, State-space, State-dependent coupling, fMRI

## Abstract

Dynamic causal modelling (DCM) was introduced to study the effective connectivity among brain regions using neuroimaging data. Until recently, DCM relied on deterministic models of distributed neuronal responses to external perturbation (e.g., sensory stimulation or task demands). However, accounting for stochastic fluctuations in neuronal activity and their interaction with task-specific processes may be of particular importance for studying state-dependent interactions. Furthermore, allowing for random neuronal fluctuations may render DCM more robust to model misspecification and finesse problems with network identification. In this article, we examine stochastic dynamic causal models (sDCM) in relation to their deterministic counterparts (dDCM) and highlight questions that can only be addressed with sDCM. We also compare the network identification performance of deterministic and stochastic DCM, using Monte Carlo simulations and an empirical case study of absence epilepsy. For example, our results demonstrate that stochastic DCM can exploit the modelling of neural noise to discriminate between direct and mediated connections. We conclude with a discussion of the added value and limitations of sDCM, in relation to its deterministic homologue.

## Graphical abstract

Stochastic DCM analysis of fMRI time series: the route to absence seizure.

**Figure:**
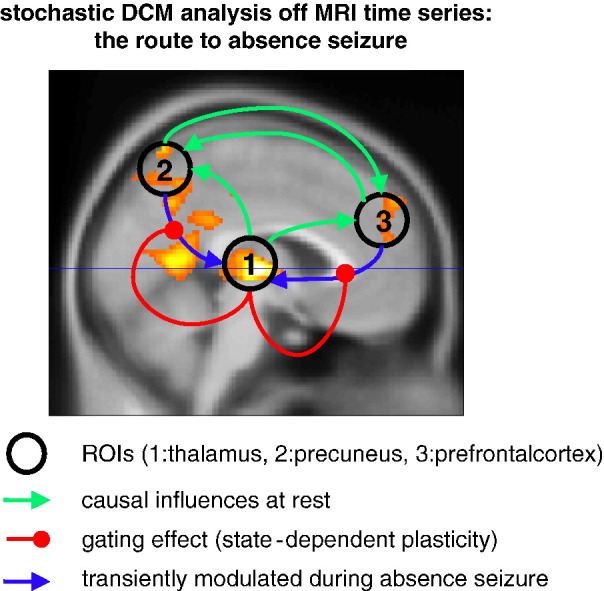

**Highlights:**

► Theory and numerics of the impact of neural noise on network identification. ► Pros and cons of modelling neural noise with DCM. ► Quantitative modelling of state-dependant plasticity during epileptogenic activity.

## Introduction

This article is about modelling distributed neuronal activity in the brain that is mediated by connections among different brain areas or sources. Brain connectivity can be characterised in three distinct ways: (i) structural connectivity, (ii) functional connectivity and (iii) effective connectivity (e.g., Sporns, 2007). Structural connectivity; i.e. the anatomical layout of axons and synaptic connections, determines which neural units interact directly with each other (e.g., Zeki and Shipp, 1988). Functional connectivity subsumes non-mechanistic (often whole-brain) descriptions of statistical dependencies among measured neuroimaging time series (e.g., Greicus et al., 2002). Finally, effective connectivity refers to causal effects; i.e. the directed influence that system elements exert on each other (Friston et al., 2007a). This article is concerned with the analysis of effective connectivity using dynamic causal modelling (DCM, see Daunizeau et al., 2010 for a recent review).

DCM is used widely in the neuroimaging community and has proven useful in disclosing neurobiological mechanisms underlying, for example, associative learning (Den Ouden et al., 2010), speech comprehension (Leff et al., 2008) or motivational processes (Schmidt et al., in press). The DCM framework has two main components: biophysical modelling and statistical data analysis (probabilistic inference). As with any modelling endeavour, both components rests on the plausibility of the modelling assumptions and the simplifications that modelling inevitably entails (e.g., Roebroeck et al., 2011). In particular, classical DCM implementations, particularly for fMRI, have assumed deterministic neuronal processes and ignore random fluctuations of physiological states. Stochastic DCM departs from deterministic DCM in that it allows for unknown (random) fluctuations or innovations to drive the neural system, in addition to the known (deterministic) experimental stimulation or control. In theory, accounting for random effects on the system's dynamics allows us to cope with imperfect model assumptions and non-specific physiological perturbations (Valdés-Sosa et al., 2011). However, it is not trivial to determine the impact of neural noise on system dynamics, particularly in the presence of nonlinearities. Furthermore, the identification of stochastic nonlinear dynamical systems is notoriously difficult (Kloeden and Platen, 1999).

We have previously proposed variational Bayesian approaches to the identification of stochastic nonlinear dynamical systems (Daunizeau et al., 2009; Friston et al., 2008, 2010). In addition to the model evidence, these approaches furnish an approximation to both the posterior densities of model parameters and states. Although these schemes were evaluated on benchmark stochastic nonlinear dynamical systems, they were not systematically evaluated in relation to the specific challenges that effective connectivity analyses face in practice. For instance, one of the main difficulties for these schemes is to identify the respective contributions of between-region coupling and local random fluctuations. Thus, one has to carefully consider the net advantages (or disadvantages) of introducing state noise in the generative model, and to what extent these advantages depend upon nonlinearities and signal-to-noise ratio. For example, can the modelling of neural noise improve network identification? These pragmatic questions address the feasibility and added value of stochastic dynamic causal modelling of neuroimaging data.

Recent studies have reported effective connectivity analyses using stochastic DCM. For example, Friston et al. (2011) show how stochastic DCM can be used in combination with post-hoc model comparison (Friston and Penny, 2011) to explore large model spaces. Li et al. (2011) examined several methodological issues raised by stochastic DCM (e.g., comparison of different methods for inversion of stochastic DCMs): however, we did not evaluate the relative performance of deterministic and stochastic DCMs in the absence and presence of neuronal state noise. The aim of this paper is to provide a systematic investigation of how stochastic DCM compares to deterministic DCM, in terms of the relative statistical efficiency of parameter estimation and model comparison — and ask whether the modelling of neural noise can improve network identification.

This paper comprises three sections. In the first, we present the basic elements of stochastic dynamic causal models, and consider their properties, in relation to their deterministic variants. This section serves to illustrate the dynamic repertoire of stochastic systems, and identifies non-trivial stochastic phenomena that could be expressed in measured neuroimaging time series. We then ask whether these are accurately captured by probabilistic inference, using both simulation series and empirical data. The second section serves to evaluate the face validity of stochastic DCM. More precisely, we examine the two main lines of inference in DCM; namely, parameter estimation and model comparison — the latter being evaluated in the context of discriminating direct versus mediated (indirect) connections. The focus here is on an extensive comparison with deterministic DCM, across a wide range of experimental conditions. In the third section, we turn to an empirical demonstration and apply DCM to data from an fMRI study of an epileptic patient. Our objective was to illustrate the added value of stochastic DCM in a context where (deterministic) experimental control is lacking. We close with a discussion of the implications of our findings in the final section.

## List of acronyms

The following acronyms will be used throughout this manuscript

s/d DCM: stochastic/deterministic dynamic causal modelling
VBL: Variational Bayes under the Laplace assumption
MSE: mean squared error
MCB: mean confidence bias
LBF: log-Bayes factor
PFC: prefrontal cortex
GSW: generalized spike-and-wave

## Stochastic dynamic causal modelling: theory and methods

In this section, we describe a broad class of hierarchical generative models, which we call stochastic dynamic causal models. These combine nonlinear stochastic differential equations governing the evolution of hidden-states and a nonlinear observer function, to provide a nonlinear state-space model of data. This section describes stochastic DCMs and their properties, in relation to their deterministic variant. In the next section, we compare stochastic and deterministic DCM with respect to parameter estimation and model comparison.

### The generative model

**Fig. 1:**
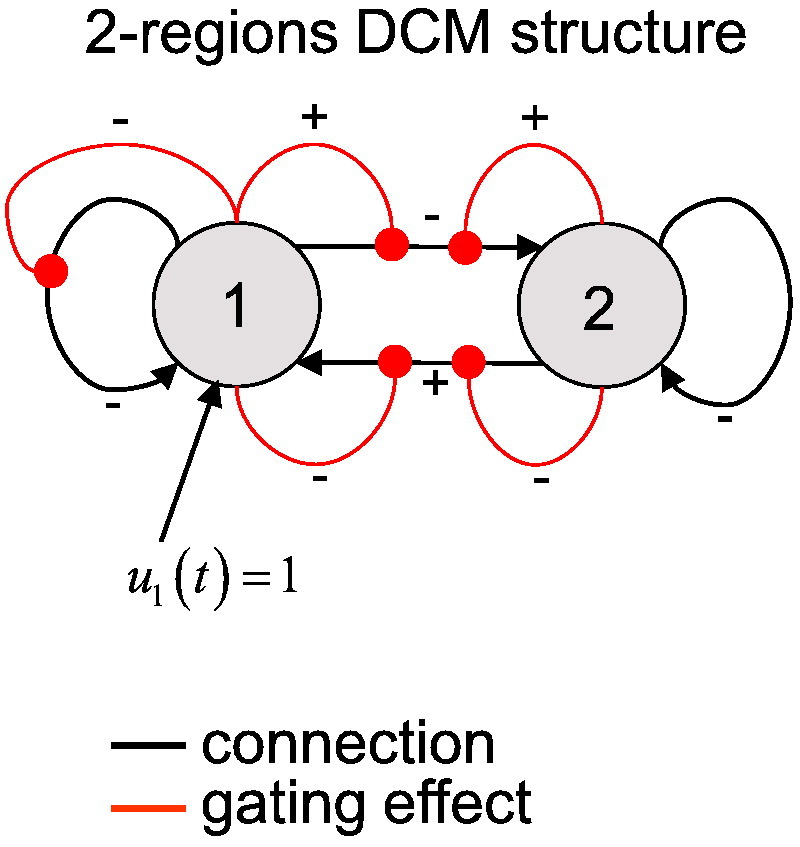
Non quasi-deterministic DCM example: network structure. This network comprises two regions, equipped with full state–state coupling (black connections) and almost full nonlinear gating effects (red connections). In addition, node 1 receives tonic (constant) input of unit magnitude.

All variants of DCMs are based on “generative models”; i.e. a probabilistic and quantitative description of the mechanisms by which observed data are generated. Typically, both hemodynamic (fMRI) and electromagnetic (EEG/MEG) signals arise from a network of functionally segregated sources (neuronal populations or cortical areas). This network can be thought of as a *directed graph*, where sources correspond to *nodes* and conditional dependencies among the hidden states of each node are mediated by effective connectivity (*edges*). The generative model *m* of a stochastic DCM rests on two causal equations:

• The *evolution equation* describes how experimental manipulations (*u*) and stochastic input (*ϖ*) influence the dynamics of hidden (neuronal) states (*x*) of the system. The Langevin form of the evolution equation of stochastic DCM can be written as follows:
  (1) $$\dot{x}=f\left(x,u,\theta \right)+\varpi \text{,}$$
  where $\dot{x}$ is the rate of change of the system's states *x*, *θ* is a set of unknown evolution parameters and *ϖ* is a stochastic (time-dependent) process that is not experimentally controlled. We will refer to *ϖ* as state noise. In this formulation, *f* summarizes the deterministic biophysical mechanisms that are parameterized by *θ* and underlie the temporal evolution of *x*; hence the name *evolution function*. The structure of the evolution or state equation determines both the presence and absence of edges in the graph and how these influence the dynamics of the system's states (see Fig. 1).
• The *observation equation* is a static nonlinear mapping from the system's hidden states (*x*) to experimental measures (*y*):
  (2) $$y=g\left(x,\varphi \right)+\varepsilon \text{,}$$
  where *g* is the *observation function*, *φ* is a set of unknown observation parameters and *ε* are (time-dependent) model residuals. We will refer to *ε* as the measurement noise.

Critically, neither the states nor the parameters of the generative model are known. Hence, additional assumptions about both the state and the measurement noise are needed to fully specify the generative model. If we assume that these processes are analytic, then the covariance of *generalized* measurement noise $\tilde{\varepsilon }={\left[\varepsilon ,\dot{\varepsilon },\ddot{\varepsilon },\ldots \right]}^{T}$ is well defined; similarly for the generalized state noise $\tilde{\varpi }=\left[\varpi ,\dot{\varpi },\ddot{\varpi },\ldots \right]$. This means we can parameterise the random part of the generative model in terms of the covariance (inverse precision) of the noise terms. Under local linearity assumptions, the above generative model can be expressed in generalized coordinates of motion, as follows:

(3) $$\begin{array}{l} \tilde{y}=\tilde{g}\left(\tilde{x},\theta \right)+\tilde{\varepsilon }\\ D\tilde{x}=\tilde{f}\left(\tilde{x},\tilde{u},\varphi \right)+\tilde{\varpi } \end{array}\text{,}$$

where D is a temporal derivative matrix operator and $\tilde{f}$ and $\tilde{g}$ are generalized evolution and observation functions (Friston et al., 2008):

(4) $$D=\left[\begin{array}{llll} 0 & 1 & & \\ & 0 & 1 & \\ & & \ddots & \ddots \end{array}\right]\;\text{,}\;\tilde{f}\left(\tilde{x},\theta ,u\right)=\left[\begin{array}{c} f\\ \frac{\partial f}{\partial x}\dot{x}+\frac{\partial f}{\partial u}\dot{u}\\ \vdots \end{array}\right]\;\text{,}\;\tilde{g}\left(\tilde{x},\varphi \right)=\left[\begin{array}{c} g\\ \frac{\partial g}{\partial x}\dot{x}\\ \vdots \end{array}\right]\text{.}$$

In addition to Eqs. (3) and (4), priors on the generalized state noise $\tilde{\varpi }$ and residuals $\tilde{\varepsilon }$, as well as on the evolution and observation parameters *θ* and *φ*, are required to fully specify the generative model. Without loss of generality, these are usually taken to be zero-mean Gaussian random variables:

(5) $$\begin{array}{l} p\left(\tilde{\varpi }|m\right)=N\left(0,\Psi_{\varpi }\right)\\ p\left(\tilde{\varepsilon }|m\right)=N\left(0,\Psi_{\varepsilon }\right)\\ p\left(\theta |m\right)=N\left(0,\Psi_{\theta }\right)\\ p\left(\varphi |m\right)=N\left(0,\Psi_{\varphi }\right) \end{array}$$

where *Ψ*_•_ are either fixed or unknown covariance matrices. In the latter case, *Ψ*_•_ is generally taken to be a mixture of covariance components, with unknown hyperparameters *λ*_•_, i.e. $\Psi_{•}=\sum_{i}\lambda_{•i}\;Q_{•i}$, where the *Q*_•_ are covariance component matrices^1^ (see e.g., Friston et al., 2008). Hierarchical priors *p*(*λ*|*m*) on hyperparameters are thus required for estimating noise covariances. We will see examples of this hierarchical structure later.

Statistical assumptions about the higher order motion of the generalized state noise $\tilde{\varpi }$ implicitly specify its degree of smoothness. Interested readers will find a theoretical motivation for using analytical state noise in the context of studying brain dynamics in Friston et al. (2011). Note that standard stochastic differential equations (and related Ito calculus) rely upon *ϖ* being a mixture of Wiener processes, which are non differentiable functions of time (Kloeden and Platen, 1999). This corresponds to a special case of generalized state noise $\tilde{\varpi }$ whose high order motion $\dot{\varpi },\ddot{\varpi },\ldots$ have infinitely high prior variance (unbounded roughness). Within the above class of generalized state-space models, standard Wiener based state-space models are thus the *least* informed or constrained. Within the context of biological time series analysis (such as neuroimaging), using such non-analytical or rough state noise might thus be unrealistic. However, in this paper, we will focus on standard state space models under Markovian (Wiener) assumptions, because these are the most established and ubiquitous models (Valdes-Sosa et al., 2011). We will return to the properties of generalized state noise in the discussion.

### What does neural noise add to DCM?

Adding stochastic forcing (random state noise or fluctuations) to a deterministic system can have intriguing dynamical consequences. What follows is a summary of the most salient differences between stochastic and deterministic systems. We sacrifice a little mathematical rigour for simplicity, in the hope of providing a didactic overview of the role of neural noise in DCM.

The effect of random fluctuations on the dynamical behaviour of a system depends upon the nature of the system itself: it turns out that some systems are so simple that any trajectory of states – in their stochastic form – resembles the deterministic path when the state noise variance is sufficiently small (in the limit *Ψ*_*ϖ*_ → 0). Such systems are called *quasi-deterministic systems* (Tropper, 1977), because their dynamical behaviour can be understood from that of their deterministic variant. Systems whose deterministic variant is sufficiently insensitive to initial conditions (e.g., linear systems) are quasi-deterministic.

In contrast, in systems that are not quasi-deterministic, a trajectory can diverge from its deterministic evolution, even at the limit of very small noise. Here, the impact of stochastic noise is the result of the tendency of small perturbations that are normal (orthogonal) to the deterministic path to decay or to grow (in locally stable or unstable parts of state-space respectively). This can be seen as follows. Let *x*_0_(*t*) be a solution of the deterministic version of Eq. (1), and *x*(*t*) = *x*_0_(*t*) + *δx*(*t*) be a perturbed trajectory. If the initial perturbation *δx*(0) is sufficiently small its evolution can be described by the linearised equation:

(6) $$\begin{array}{c} \frac{d}{dt}\delta x=J\delta x\\ J=\frac{\partial f}{\partial x} \end{array}\text{.}$$

**Fig. 2:**
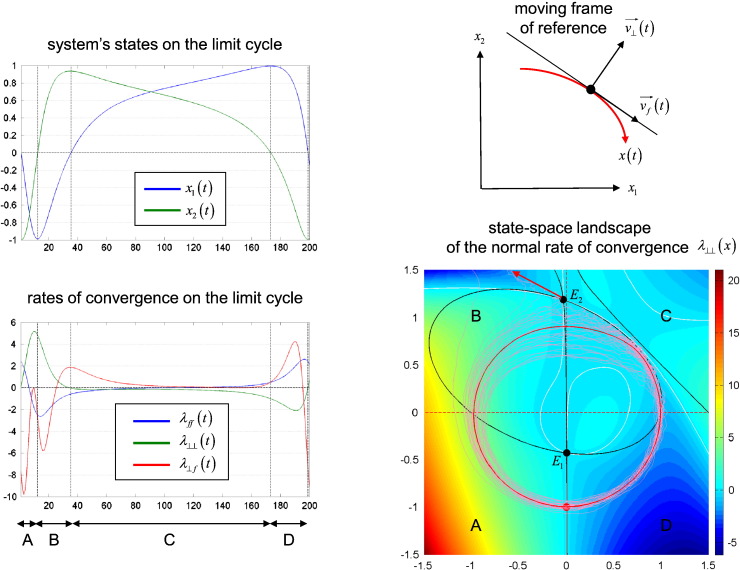
Non quasi-deterministic DCM example: state-space analysis. This figure summarizes the local stability structure of the network depicted in Fig. 1, which possesses a limit cycle centred on the origin. Upper-left: this panel shows one cycle of the deterministic orbit of the two states of the network (blue: *x*_1_(*t*), green: *x*_2_(*t*)), as a function of time. One can distinguish four phases or quadrants: A: *x*_1_ ≤ 0 ∩ *x*_2_ ≤ 0, B: *x*_1_ ≤ 0 ∩ *x*_2_ ≥ 0, C: *x*_1_ ≥ 0 ∩ *x*_2_ ≥ 0 and D: *x*_1_ ≥ 0 ∩ *x*_2_ ≤ 0. Upper-right: schematic of the moving frame of reference. The reference solution (orbit *x*(*t*) on the limit cycle) is in red. At some arbitrary point on this orbit, ${\vec{v}}_{f}\left(t\right)$ indicates the direction tangential to the flow, and ${\vec{v}}_{⊥}\left(t\right)$ indicates the transverse or normal direction. Lower-left: dynamics of the three elements (exponents) of the stability matrix *J* * (see Eq. (10); *λ*_*ff*_(*t*): blue, *λ*_⊥ ⊥_(*t*): green, *λ*_⊥ *f*_(*t*): red). Quadrants A and B are such that *λ*_⊥ ⊥_ ≥ 0; i.e. the system is locally unstable in the transverse direction, and quadrants C and D are such that *λ*_⊥ ⊥_ ≤ 0; i.e. the system is locally stable in the transverse direction. Lower-right: profile of the transverse divergence *λ*_⊥ ⊥_ over the state-space. The white solid curves show the level set *λ*_⊥ ⊥_ = 0. The black solid curves show the nullclines of the system, respectively ${\dot{x}}_{1}=0$ and ${\dot{x}}_{2}=0$. They intersect at two points *E*_1_, which is an unstable node, and *E*_2_, which is a saddle point. The solid red circle shows the limit cycle of the network, and pink traces are sample paths in the presence of state noise. The red arrow shows the repelling direction of the saddle-point *E*_2_, along which some sample paths were expelled from the basin of attraction of the limit cycle.

The Jacobian *J* measures the local stability of the path: the local motion of the perturbed path is determined by the matrix *J*, which mixes and rescales the perturbation *δx*. Of particular importance is the direction of the perturbation — if the perturbation is tangential to the flow, it will not change the deterministic motion. Consider the projection of a perturbation onto a frame of reference that moves with the path: *δx* * = *Uδx*, where *U* is the projection matrix and *δx* * is the perturbation in the moving frame of reference (see Fig. 2). The resulting perturbation dynamics are:

(7) $$\begin{array}{l} \frac{d}{dt}\delta x*=J*\delta x*\\ \;J*=UJU^{-1}+\frac{dU}{dt}U^{-1}\\ \; \end{array}$$

where *J* * is the local stability matrix in the moving frame of reference. For two-dimensional systems, the local stability matrix *J* * can be derived analytically and has the following form (see Equation 14 in Ali and Menzinger, 1999):

(8) $$\begin{array}{l} J*=\frac{1}{\sqrt{f^{T}f}}\left[\begin{array}{c} f_{1}^{2}J_{22}+f_{2}^{2}J_{11}+2f_{1}f_{2}\left(J_{12}+J_{21}\right)\\ 0 \end{array}\;\begin{array}{c} \left(f_{1}^{2}-f_{2}^{2}\right)\left(J_{12}+J_{21}\right)+2f_{1}f_{2}\left(J_{11}+J_{22}\right)\\ f_{1}^{2}J_{22}+f_{2}^{2}J_{11}-2f_{1}f_{2}\left(J_{12}+J_{21}\right) \end{array}\right]\\ \text{ =}\left[\begin{array}{c} \lambda_{\mathrm{ff}}\\ 0 \end{array}\begin{array}{c} \lambda_{⊥f}\\ \lambda_{⊥⊥} \end{array}\right] \end{array}$$

where *λ*_⊥ ⊥_ is the rate of exponential divergence (exponent) normal or transverse to the flow *f*(*x*) and *λ*_*ff*_ is the tangential exponent. Since *λ*_*f* ⊥_ = 0, any perturbation that is initially aligned with the flow will remain tangential, accelerating (*λ*_*ff*_ > 0) or decelerating (*λ*_*ff*_ < 0) the unperturbed motion. Note that this holds for higher-dimensional systems (Ali and Menzinger, 1999); that is, the local stability matrix always has the form of Eq. (8). On the other hand, the transverse component of the perturbation will be partially projected back onto the tangential motion (with amplitude *λ*_⊥ *f*_ ≠ 0). This leads to phase resetting phenomena (see Winfree, 1980) such as phase advance and retard when *λ*_⊥ *f*_ > 0 and *λ*_⊥ *f*_ < 0 respectively. Finally, the perturbed path *x*(*t*) can diverge from *x*_0_(*t*) only if the system is locally unstable in the transverse direction; in other words, if the transverse exponent is positive (*λ*_⊥ ⊥_ ≥ 0). If, in addition, the deterministic variant of the system has more than one equilibrium point, it may then deviate from its deterministic variant and escape from the local basin of attraction. When there are several *stable* attractors (as in multistable systems) the system can switch from one attractor to another (cf. Fig. 17 in Daunizeau et al., 2009). Such transitions are the hallmark of (non quasi-deterministic) stochastic dynamical systems and can dramatically extend the system's dynamical repertoire, leading to non trivial phenomena such as stochastic resonance or hysteresis (Berglund and Gentz, 2005; McDonnel and Abbot, 2009).

Consider these issues in the context of the evolution function *f*^(*n*)^ of neural states *x*^(*n*)^ that is used in nonlinear DCM for fMRI (Stephan et al., 2008):

(9) $$f^{\left(n\right)}\left(x^{\left(n\right)},u,\theta \right)=\underset{linear}{\underset{︸}{Ax^{\left(n\right)}+\sum_{i}u_{i}B^{\left(i\right)}x^{\left(n\right)}+Cu}}+\underset{gating}{\underset{︸}{\sum_{j}x_{j}^{\left(n\right)}D^{\left(j\right)}x^{\left(n\right)}}}\text{.}$$

Here, each (neural) state *x*_*i*_^(*n*)^(*t*) can be regarded as the amplitude of the slowest eigenmode of an (immensely high dimensional) neural system in the *i*th region of interest (see Friston et al., 2011). In this context, Eq. (9) is simply a second order Taylor expansion of the underlying unknown evolution function (Friston et al., 2003; Stephan et al., 2007). First, we note that stochastic DCMs (for fMRI) that do not include nonlinear interactions among the states are ergodic and quasi-deterministic. Their ergodic property means that these systems will eventually reach a stationary distribution (the so-called “sojourn distribution”); in other words, all trajectories will exhibit fluctuations around their fixed point.^2^ The frequency spectrum of these fluctuations can be derived from the Laplace transform of the system, which depends upon the connectivity matrix *A* and the neural noise level *Ψ*_*ϖ*_^(*n*)^ (for example, see Moran et al., 2007 for an application of DCM to steady-state EEG/MEG data). Heuristically, the amplitude of these oscillations is inversely proportional to their frequency. This is because the states experience small deviations from zero exponentially more often than large deviations. In linear DCMs, the local stability matrix is constant over state-space (the Jacobian does not depend upon the states). This means that the effect of neural noise is homogeneous over state-space.

However, stochastic DCMs that include nonlinear interactions (such as those in Eq. (9)) are less trivial: the stationary distribution of these systems is likely to be multimodal. This precludes a quasi-deterministic system. Furthermore, the effect of noise is not constant across state-space, since the Jacobian *J*^(*n*)^ = *A* + ∑ _*i*_*u*_*i*_*B*^(*i*)^ + 2 ∑ _*j*_*x*_*j*_^(*n*)^*D*^(*j*)^ is a function of the states. We will illustrate this with a simple two-region (2D) DCM, whose connectivity structure is shown in Fig. 1.

This network exhibits almost full nonlinearities (i.e., almost all *D* coefficients are non-zero) and has tonic input to region 1. Fig. 2 summarizes the state-dependent effect of noise on the system.

It turns out that this system has a limit cycle that resembles the unit circle, surrounding an unstable node *E*_1_. This means that the limit cycle is approached by any trajectory starting inside it, which will spiral out, being repelled by *E*_1_. In addition, the system has another equilibrium point *E*_2_, which is a saddle-node outside the limit cycle. This means that deviations of the trajectory sufficiently far from the attractor can cause trajectories to diverge. However, this can only happen in some regions of the state-space. Typically, once the system has entered the limit cycle, the system can only deviate from it when the limit cycle is unstable in the transverse direction (c.f. Eqs. (7) and (8)). The transverse exponent shows this very clearly: The dispersion around the limit cycle is much higher in regions where *λ*_⊥ ⊥_ ≥ 0 (e.g., quadrants A and B), because any transverse perturbation is amplified. The distorted path is thus more likely to escape the limit cycle's basin of attraction. This happens in quadrant B, whenever the distorted path gets close enough to the saddle point *E*_2_ (along its attracting direction), which then repels it (along its repelling direction). In contrast, the flow is almost zero in quadrant C; here, the deterministic path is very slow, because the elements of the stability matrix are small, except for the non-diagonal divergence *λ*_⊥ *f*_ > 0. This causes the trajectories to wander around the deterministic trajectory; note how the noise “wraps” itself around the deterministic trajectory. Finally, the system accelerates in quadrant D, which is locally stable since any transverse perturbation is heavily damped (*λ*_⊥ ⊥_ ≪ 0 and *λ*_⊥ *f*_ ≫ 0: note the small dispersion around the attractor). In brief, this system can be considered (locally) quasi-deterministic in quadrants C and D, where the transverse exponent is negative. But it is definitely not quasi-deterministic in quadrant A or B, where small perturbations can lead the trajectories to diverge exponentially from the deterministic path.

In brief, stochastic systems whose deterministic variant has more than one fixed point will not be quasi-deterministic. In the context of DCM for fMRI data, nonlinearities in the evolution function arise from the interaction or gating effects (nonzero *D*^(*j*)^ matrices in Eq. (9)) and from the hemodynamic equations that model the coupling between neuronal activity and the fMRI signal. However, this hemodynamic model, which is described in the next section, has only one stable fixed point. Its stochastic extension is thus quasi-deterministic. Therefore, non quasi-deterministic behaviour in the system only occurs when (neural) nonlinear gating effects are considered. We will look at the implications of non quasi-deterministic dynamics for statistical model identification in detail below.

### A note on hemodynamics

In addition to the neural evolution function given in Eq. (9), DCM for fMRI requires the specification of an additional set of hemodynamic states that couple neural dynamics to observed BOLD signal changes:

(10) $$f^{\left(h\right)}\left(x\right)=\left[\begin{array}{c} x^{\left(n\right)}-\kappa_{s}x_{1}^{\left(h\right)}-\kappa_{f}\left(e^{x_{2}^{\left(h\right)}}-1\right)\\ x_{1}^{\left(h\right)}e^{-x_{2}^{\left(h\right)}}\\ \frac{1}{\tau_{0}}\left(e^{x_{2}^{\left(h\right)}}-e^{x_{3}^{\left(h\right)}/\alpha }\right)e^{-x_{3}^{\left(h\right)}}\\ \frac{1}{\tau_{0}}\left(\frac{e^{x_{2}^{\left(h\right)}-x_{4}^{\left(h\right)}}\left(1-{\left(1-E_{0}\right)}^{e^{-x_{2}^{\left(h\right)}}}\right)}{E_{0}}-e^{\left(1-\alpha \right)x_{3}^{\left(h\right)}/\alpha }\right) \end{array}\right]$$

where *x*^(*n*)^ is a regional neural activity, whose dynamics is given in Eq. (9). Eq. (10) expresses changes in hemodynamic states *x*^(*h*)^, as a response to a neural perturbation *x*^(*n*)^. Finally, one has to specify the observation mapping (Eq. (2)) from hemodynamic states *x*^(*h*)^ to observed local BOLD changes *y*:

(11) $$g\left(x\right)=V_{0}\left(4.3\nu_{0}E_{0}TE\left(1-e^{x_{4}^{\left(h\right)}}\right)+\varepsilon_{0}r_{0}E_{0}TE\left(1-e^{x_{4}^{\left(h\right)}-x_{3}^{\left(h\right)}}\right)+\left(1-\varepsilon_{0}\right)\left(1-e^{x_{3}^{\left(h\right)}}\right)\right)\text{.}$$

We refer the reader to Stephan et al. (2007) for details about the form of Eqs. (10) and (11).

Unless stated explicitly, the values of the hemodynamic parameters (used for both simulations and model inversion) are given in Table 1.

In the hemodynamic model, blood flow (*x*_2_^(*h*)^), blood volume (*x*_3_^(*h*)^) and deoxyhemoglobin content (*x*_4_^(*h*)^) are log-transformed to enforce positivity (c.f. Appendix in Stephan et al., 2008). The system in Eq. (10) has only one (stable) equilibrium point at *x*^(*h*)^ = [0, 0, 0, 0]^*T*^, which makes it weakly nonlinear. This implies that – when integrated over time – Eqs. (10) and (11) behave much like a second-order linear convolution (Friston et al., 2003), whose impulse response function would be very similar in shape to the canonical hemodynamic response function used for classical SPM data analysis (Friston et al., 2000, 2007a). One may think that, since the stochastic variant of the haemodynamic model is quasi-deterministic (since it has only one stable equilibrium point), one could accommodate its biophysical inaccuracies through the introduction of hemodynamic state noise *ϖ*^(*h*)^. However, practical experience shows that model inversion is confounded by hemodynamic state noise, because any BOLD signal change can be explained by hemodynamic state noise, effectively ‘shunting’ an explanation of the data in terms of neural changes. We therefore have to ensure that this explaining away is a priori implausible. In what follows, we partition the state noise prior into neural (*Ψ*_*ϖ*_^(*n*)^) and hemodynamic (*Ψ*_*ϖ*_^(*h*)^) components, with the constraint that: *Ψ*_*ϖ*_^(*n*)^ = 10^2^*Ψ*_*ϖ*_^(*h*)^. This a priori constraint favours an explanation of measured BOLD signals in terms of neuronal processes, over a competing explanation resting on hemodynamic noise. We will return to this in the discussion.

### Approximate probabilistic (Bayesian) inference

Inverting the generative model above means inferring the system's parameters (e.g., matrices {*A*, *B*^(*i*)^, *C*, *D*^(*j*)^} ∈ *θ* in Eq. (9)) from one trajectory of hidden physiological states, which we observe through a (potentially nonlinear) transformation with discretely sampled measurements. Priors on the state noise (Eq. (5)) specify our assumptions about the magnitude of state noise. In many instances, we will believe a priori that the state noise is small. In this case, the Bayesian inversion of the generative model will try to explain as much of observed signal variance as possible in terms of deterministic flow, by minimizing state noise (but see below).

The measured data *y* are a nonlinear function of the unknown model variables *ϑ* = {*x*, *θ*, *φ*}. This implies that the high-dimensional integrals required for parameter estimation and model comparison cannot be evaluated analytically. Furthermore, it is computationally costly to evaluate them using numerical brute force or Monte Carlo sampling schemes. This is why several *variational Bayesian* schemes have been derived for approximate probabilistic inversion of stochastic DCM (for details, see Daunizeau et al., 2009a; Friston et al., 2008, 2010). In brief, variational schemes optimize an approximation to the model evidence *p*(*y*|*m*) and posterior density *p*(*ϑ*|*y*, *m*). This is done by noting that the log model evidence can be decomposed as follows:

(12) $$\ln p\left(y|m\right)=F\left(q\right)+D_{\mathrm{KL}}\left(q\left(\vartheta \right);p\left(\vartheta |y,m\right)\right)\text{,}$$

where *q*(*ϑ*) is an arbitrary density over the model parameters, *D*_*KL*_ is the Kullback–Leibler divergence, and the *free energy F*(*q*) is defined as:

(13) $$F\left(q\right)={\langle \ln p\left(\vartheta |m\right)+\ln p\left(y|\vartheta ,m\right)-\ln q\left(\vartheta \right)\rangle }_{q}\text{,}$$

where the expectation 〈 · 〉_*q*_ is taken under *q*. From Eq. (12), maximizing the functional *F*(*q*) with respect to *q* minimizes the Kullback–Leibler divergence between *q*(*ϑ*) and the exact posterior *p*(*ϑ*|*y*, *m*). This decomposition is complete in the sense that if *q*(*ϑ*) = *p*(*ϑ*|*y*, *m*), then *F*(*q*) = ln *p*(*y*|*m*). Typically, the iterative maximization of free energy proceeds under the Laplace approximation, where the approximate posterior *q*(*ϑ*) ≈ *p*(*ϑ*|*y*, *m*) is assumed to have a Gaussian form (see Friston et al., 2007a, 2007b). The free energy thus becomes a simple function of the first and second moments of the approximate posterior (the conditional mean and covariance):

(14) $$F_{\mathrm{Laplace}}\left(q\right)=\ln p\left(\mu |m\right)+\ln p\left(y|\mu ,m\right)+\frac{1}{2}\log\left|\Sigma \right|+\frac{1}{2}n_{\vartheta }\log2\pi \text{.}$$

Here, *n*_*ϑ*_ is the number of unknown variables in the generative model, and {*μ*, Σ} ≜ {*E*_*q*_[*ϑ*], *V*_*q*_[*ϑ*]} are the *n*_*ϑ*_ × 1 mean and *n*_*ϑ*_ × *n*_*ϑ*_ covariance of the approximate posterior density *q*(*ϑ*). We will refer to this Variational Bayesian scheme under the Laplace approximation as Variational Laplace or the VBL approach.

In essence, VBL approaches to the identification of stochastic and deterministic systems are identical. However, including state noise in Eq. (1) induces a number of challenges and advantages for statistical inference, which we detail below. Briefly, these include:

• A considerable increase in the number of unknown (hidden) variables. One consequence of this is that the inference becomes increasingly sensitive to priors, because there is less information in the data per unknown variable.
• Activity in any node of the network can now be explained in two ways: either it is due to the experimental input *u*, or it is due to random fluctuation *ϖ*. This can induce a potential indeterminacy between connectivity parameters (that propagate *u* through the network) and inferred state noise.
• When considering nonlinear gating effects, the small noise limit of the generative model *m* is *not* the corresponding deterministic DCM (c.f. non quasi-deterministic systems; see above). Since the trajectories of such systems cannot be predicted from their deterministic variant, the identification of nonlinear systems may improve if noise is modelled properly.

In the next section, we compare deterministic and stochastic DCMs in terms of parameter estimation and model comparison, using numerical Monte-Carlo simulations. We pay particular attention to the identification of mediated versus direct connections, because most of the above comments apply to this case. In the subsequent section, we provide an example of the sort of inference that can be made when the system is not experimentally controlled — using fMRI data required from a patient during absence (“petit mal”) seizures.

## Comparing deterministic and stochastic dynamic causal modelling: results

The technical details of variational Bayesian treatments of stochastic systems have been published elsewhere (Daunizeau et al., 2009a; Friston et al., 2008, 2010). In brief, these methods appear to be accurate when estimating parameters (using the approximate posterior densities) and comparing models (using the free energy bound on log model evidence). This has been established using Monte-Carlo procedures based on benchmark stochastic dynamical systems. In this section, we apply the VBL described in Daunizeau et al. (2009) to assess the added value of stochastic dynamic causal modelling, with respect to its deterministic variant, within the context of DCM for fMRI data.

For simplicity, we did not model generalised state noise; i.e. $\tilde{\varpi }=\varpi$. The impact of modelling generalised motion will be assessed in forthcoming publications (see also Li et al., 2011). To accommodate hemodynamic delays, we extended the forward pass of the variational Bayesian Kalman filter presented in Daunizeau et al. (2009). This extension is described in the Appendix A: in brief, this extension ensured that the state estimate at time *t* is conditioned upon fMRI data up to time *t* + *Δt*. We found that *Δt* = 16 s gave both efficient and robust results. In what follows, we compare deterministic and stochastic DCM with respect to parameter estimation and model comparison, using Monte-Carlo simulations.

### Monte-Carlo simulations: assessing estimation accuracy

First, we asked whether one can improve the estimation of unknown DCM variables by including state noise in the generative model. To do so, we compare the deterministic and stochastic variants of the same DCM, in terms of their ability to recover (i) network connectivity parameters, (ii) region-dependent inputs and (iii) neural state dynamics. We assume that the relative estimation accuracy of dDCM and sDCM will depend upon system or data properties, such as SNR, the presence of nonlinearities in the neural evolution function and the presence of neural fluctuations or state noise.

First, note that one can extend a deterministic DCM to accommodate unknown fluctuations by projecting state noise *ϖ*^(*n*)^ onto some known (temporal) basis functions ${\overset{⌢}{u}}_{k}$:

(15) $$\varpi_{i}^{\left(n\right)}\left(t\right)\approx \sum_{k=1}^{K}{\overset{⌢}{u}}_{k}\left(t\right){\overset{⌢}{c}}_{\mathrm{ik}}$$

where ${\overset{⌢}{c}}_{\mathrm{ik}}$ are unknown projection coefficients, which define the temporal profile of fluctuating noise *ϖ*_*i*_^(*n*)^(*t*). This gives the augmented evolution function (cf., Eq. (9))

(16) $$f^{\left(n\right)}\left(x^{\left(n\right)},u,\theta \right)=Ax^{\left(n\right)}+\sum_{i}u_{i}B^{\left(i\right)}x^{\left(n\right)}+\left[\begin{array}{cc} C & \overset{⌢}{C} \end{array}\right]\left[\begin{array}{c} u\\ \overset{⌢}{u} \end{array}\right]+\sum_{j}x_{j}^{\left(n\right)}D^{\left(j\right)}x^{\left(n\right)}$$

where $\overset{⌢}{u}\left(t\right)={\left[\begin{array}{cccc} {\overset{⌢}{u}}_{1}\left(t\right) & {\overset{⌢}{u}}_{2}\left(t\right) & \cdots & {\overset{⌢}{u}}_{K}\left(t\right) \end{array}\right]}^{\text{T}}$ comprise the temporal basis functions. Eqs. (15) and (16) mean that the problem of recovering unknown neural fluctuations *ϖ*^(*n*)^(*t*) can be transformed into a standard (deterministic) DCM parameter estimation problem. The question we address here is: if the goal is to account for unknown (unspecific) neural state noise, should we use a stochastic DCM, or a deterministic DCM extended as above?

The answer to this question depends upon the goal of model inversion. In this section, we focus on the statistical efficiency of variable estimation. The estimation error can be measured using the mean squared error (MSE):

(17) $$MSE_{\vartheta }=\frac{1}{n_{\vartheta }}\sum_{i=1}^{n_{\vartheta }}{\left(\vartheta_{i}-{\hat{\vartheta }}_{i}\right)}^{2}$$

where *ϑ* and $\hat{\vartheta }$ are the relevant vectors of simulated and estimated variables, respectively. The MSE is a standard estimation error measure, whose *a posteriori* expectation is minimized by the posterior mean. In Bayesian decision theoretic terms, this means that choosing the posterior mean as the estimator (i.e. $\hat{\vartheta }≜\mu$) is optimal with respect to squared error loss. The observed MSE thus gives us a measure of estimation accuracy.

In addition, we want to assess the quality of the posterior credible intervals; that is we want to quantify the under or over confidence of the inference. This can be done simply by noting that the posterior variance *Σ* is actually the expected estimation error:

(18) $$\begin{array}{c} E\left[MSE_{\vartheta }|y,m\right]=\frac{1}{n_{\vartheta }}\sum_{i=1}^{n_{\vartheta }}E\left[{\left(\vartheta_{i}-\mu \right)}^{2}|y,m\right]\\ =\frac{1}{n_{\vartheta }}trace\left[\Sigma \right] \end{array}$$

Eq. (18) means that on average, the estimation error should be consistent with the second-order moment of the approximate posterior *q*(*ϑ*). In other terms, if the MSE is systematically bigger (respectively, smaller) than the posterior variance, the method is over- (respectively, under) confident (Daunizeau et al., 2009a). We thus define the mean confidence bias (MCB) as follows:

(19) $$MCB_{\vartheta }=\frac{1}{n_{\vartheta }}\sum_{i=1}^{n_{\vartheta }}{\left(\vartheta_{i}-\mu_{i}\right)}^{2}-\Sigma_{\mathrm{ii}}\text{.}$$

The mean confidence bias measures the departure from the expected estimation error in Eq. (18), such that when *MCB* > 0 (resp. *MCB* < 0), inference is over confident (resp. under confident).

To estimate the accuracy and confidence bias we conducted the following series of Monte-Carlo simulations. We generated data *y* from a DCM with two regions (see below). We analyzed the simulated data using both deterministic and stochastic variants of DCM. We then compared their respective MSE and MCB on (i) the connectivity parameters, (ii) the region-dependent input and (iii) the neural states.

**Fig. 3:**
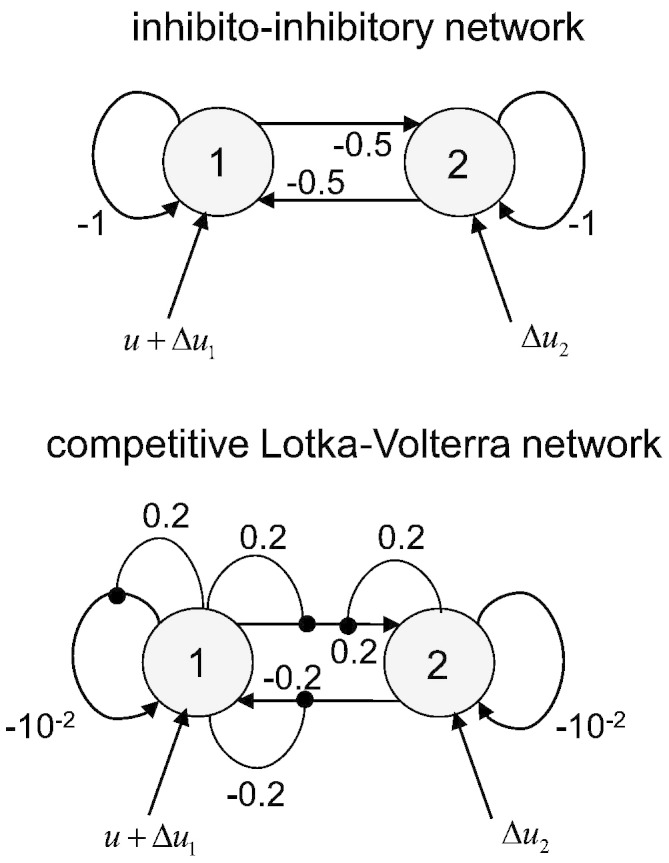
Two-region networks: linear and non-linear interactions. This figure summarizes the respective structure of networks used to simulate fMRI BOLD signal. Upper half: two regions with (linear) inhibitory–inhibitory interactions. Note that node 1 is always driven by a known input *u* (see main text). In addition, independent random fluctuations *Δu* the hidden states in both nodes. The simulated connection strengths are indicated near the appropriate arrow of the graph. Lower half: two regions in (nonlinear) competition. The connections strengths are chosen such that the ensuing system possesses a limit cycle.

Crucially, we manipulated three factors that may influence the relative estimation accuracy of deterministic versus stochastic DCM, namely:

• *SNR*: The signal-to-noise ratio was either set to 1 or to 10 dB, according to the standard definition of SNR in decibels, i.e.: *SNR* = 10 log _10_(〈*g*^2^〉/〈*ε*^2^〉), where *g* are the system observables and ε is measurement noise (see Eq. (2)).
• Δ*u*: The presence of neural fluctuations or neural noise: the simulated system was either driven by an exogenous input *u* with a boxcar structure of one minute duration — or by the same input plus some neural fluctuations Δ*u*. Here, the fluctuations Δ*u* were a random mixture of sinusoidal fluctuations and white noise, with an effective (state) SNR of 10 log _10_(〈*u*^2^〉/〈*Δu*^2^〉) ≈ 10dB.
• *NL*: The presence of nonlinearities in the neural evolution function: the simulated network was either a linear inhibitory–inhibitory system or a competitive Lotka–Volterra system (see e.g., Hofbauer and So, 1994), whose network structure includes nonlinear gating that induces a limit cycle. The two DCM network structures are shown on Fig. 3.

For each cell of this 2 × 2 × 2 factorial simulation design, we ran 24 simulations of 10 min each (with a TR of one second), leading to 192 simulated sessions in total. Each session was then analyzed given a 2 × 3 factorial model comparison with the following two factors:

• *DCM type*: Stochastic or deterministic DCM; i.e. the generative model assumed or not the presence of state noise *ϖ*. For stochastic DCM, we used loosely informative Gamma priors on the precision of state noise, i.e. *Ψ*_*ϖ*_^(*n*) − 1^ = *λ*_*ϖ*_^(*n*)^*I* with *p*(*λ*_*ϖ*_^(*n*)^|*m*) = *Ga*(1, 10^− 1^). This means that its variance is, a priori, expected to be greater than 1/30 with prior probability 0.95.
• $\overset{⌢}{u}$ *Basis functions*: The use of temporal basis function sets $\overset{⌢}{u}$ in the neural evolution function. This factor had three levels: (1) no input basis function set, (2) Fourier basis function set and (3) radial basis function (RBF) set. We used thirty two basis functions for both Fourier and RBF per region, which meant 64 additional evolution parameters ${\overset{⌢}{c}}_{\mathrm{ij}}$ (c.f. Eq. (15)).

All models used i.i.d. shrinkage priors for the neural evolution parameters (*Ψ*_*θ*_ = 10^− 1^*I*), and an uninformative (Jeffrey's) prior on the precision of observation noise; i.e. *Ψ*_*ε*_^− 1^ = *λ*_*ε*_*I* with *p*(*λ*_*ε*_|*m*) ∝ *λ*_*ε*_^− 1^. Hemodynamic parameters were fixed to their prior mean, as given in Table 1.

The estimation accuracy (MSE) and confidence bias (MCB) for each of the three sets of model variables (input, parameters and neural states) were analysed using a 2 × 2 × 2 × 3 × 2 ANOVA (within Monte-Carlo repetitions; N = 24).

**Fig. 4:**
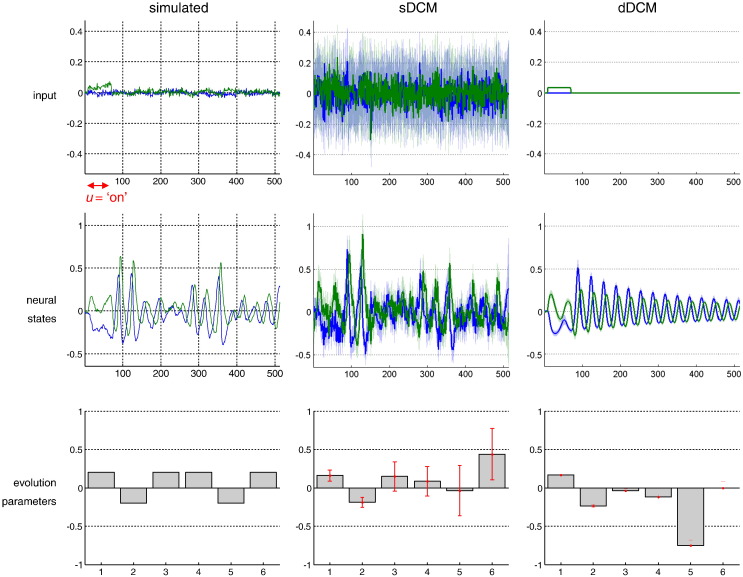
Monte-Carlo simulations: example. This figure summarizes the results of one representative simulation of the Monte-Carlo series, in the nonlinear case, with input fluctuations, at low SNR and without temporal basis functions. Left column: the simulated variables. Middle column: sDCM approximate posterior density on the simulated variables (solid line: mean, shaded area: standard deviation). Right column: dDCM approximate posterior density. Upper row: neural input to both nodes (blue: node 1, green: node 2). Middle row: trajectories of in neural states for both nodes. Lower row: neural evolution function parameters.

Fig. 4 shows exemplar results of one simulation of the nonlinear DCM, with SNR = 1 dB and perturbed input. The left column shows the simulated input (top), the dynamics of the neural states (middle) and the evolution parameters (bottom). One can see that when the experimental input *u* on region 2 (green) is ‘on’, reciprocal competition of both regions expresses itself through sustained activation (resp. deactivation) in region 2 (resp. region 1). Later, the network enters a limit cycle such that both regions are approximately *π*/2 out of phase. These transient dynamics are due to the interaction between neural noise (*Δu*) and the local stability of the limit cycle (see section “What does neural noise add to DCM?”). The middle (resp. right) column shows the posterior density on these responses for the stochastic (resp. deterministic) DCM inversion, without input basis functions $\overset{⌢}{u}$. It can be seen that both the estimation error and the confidence bias depend upon whether one uses a stochastic or a deterministic DCM. This is most evident for the neural evolution parameters, which show a higher estimation error and over confidence for the deterministic DCM.

**Fig. 5:**
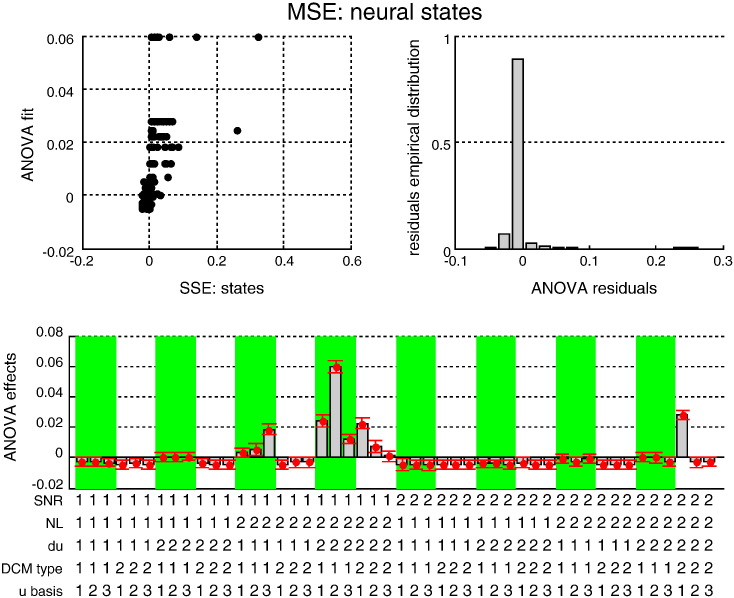
Estimation accuracy results: MSE on neural states. This figure summarizes the results of the Monte-Carlo simulations, in terms of the estimated MSE on hidden neural states as a function of the five factors of the simulation design. Upper-left: the best fit of the ANOVA (y-axis) is plotted against the actual observed MSE (x-axis) for each individual simulation. Upper right: the empirical histogram of the ANOVA residuals. Lower half: the Monte-Carlo estimate of the mean effect on MSE for each of the 48 cells of the factorial design: *SNR* = 1: low SNR, *SNR* = 2: high SNR; *NL* = 1: linear system, *NL* = 2: nonlinear system; *du* = 1: no input fluctuation, *du* = 2: input fluctuations; *DCM type* = 1: sDCM (these cells are highlighted using the green shaded areas), *DCM type* = 2: dDCM; *u basis* = 1:no input basis set, *u basis* = 2: Fourier basis set, *u basis* = 3: RBF basis set.

**Fig. 6:**
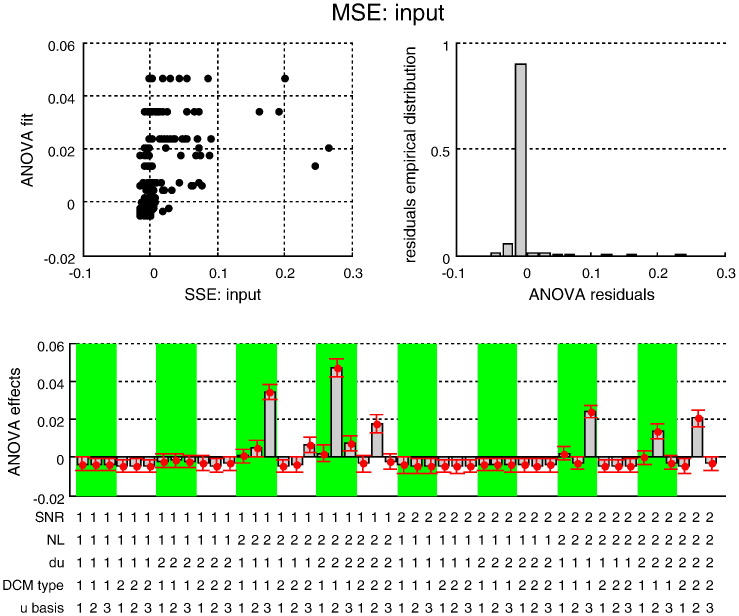
Estimation accuracy results: MSE on input. This figure summarizes the results of the Monte-Carlo simulations, in terms of the estimated MSE on the system's input as a function of the five factors of the simulation design. The figure uses the same format as Fig. 5.

**Fig. 7:**
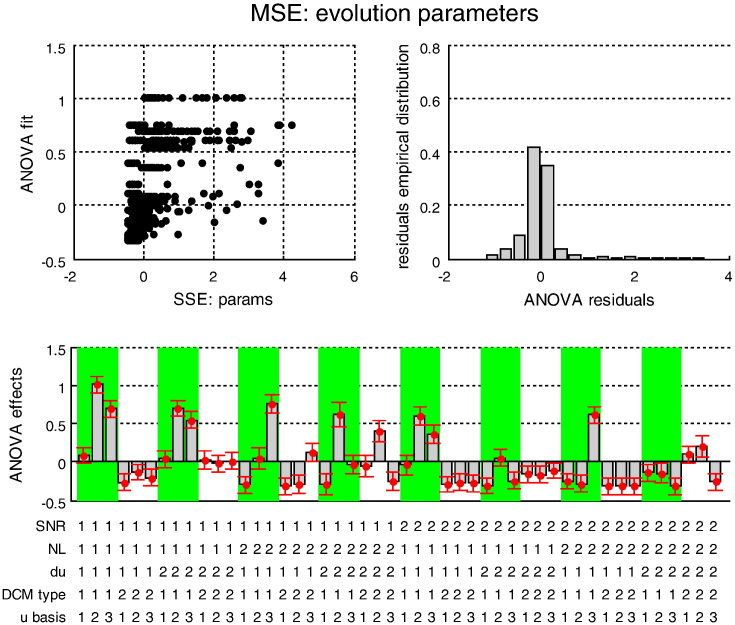
Estimation accuracy results: MSE on evolution parameters. This figure summarizes the results of the Monte-Carlo simulations, in terms of the estimated MSE on the evolution parameters as a function of the five factors of the simulation design. The figure uses the same format as Fig. 5.

**Fig. 8:**
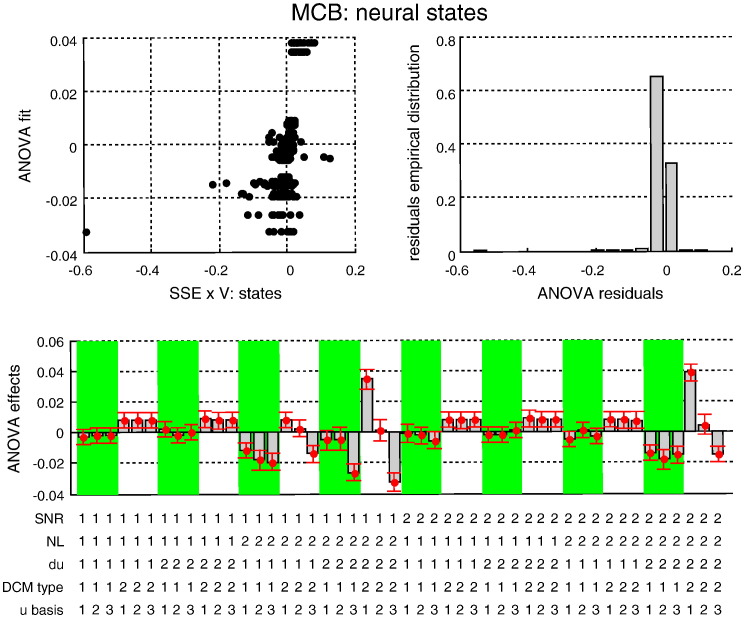
Estimation accuracy results: MCB on neural states. This figure summarizes the results of the Monte-Carlo simulations, in terms of the estimated MCB on the neural states as a function of the five factors of the simulation design. The figure uses the same format as Fig. 5.

**Fig. 9:**
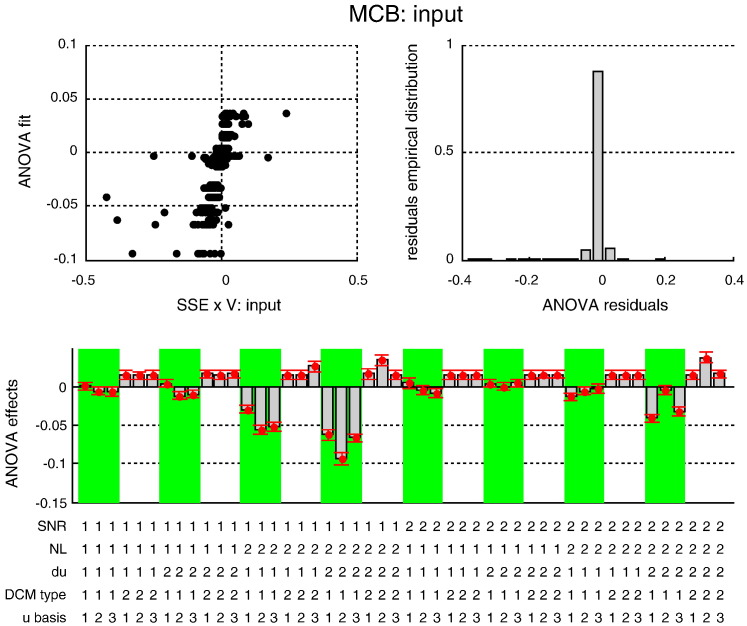
Estimation accuracy results: MCB on input. This figure summarizes the results of the Monte-Carlo simulations, in terms of the estimated MCB on the system's input as a function of the five factors of the simulation design. The figure uses the same format as Fig. 5.

**Fig. 10:**
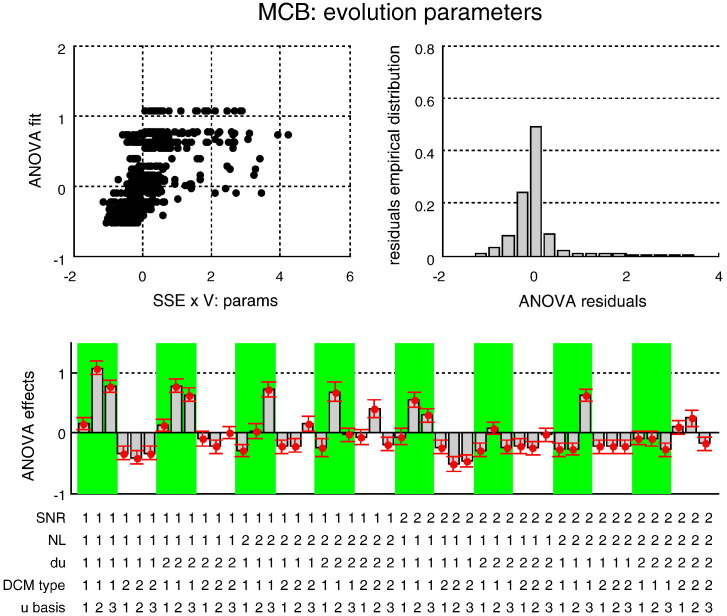
Estimation accuracy results: MCB on evolution parameters. This figure summarizes the results of the Monte-Carlo simulations, in terms of the estimated MCB on the evolution parameters as a function of the five factors of the simulation design. The figure uses the same format as Fig. 5.

Figs. 5–7 (resp. Figs. 8–10) summarize the results of the ANOVA, in terms of the estimated effects of simulation factors on the MSE (resp. MCB) for the three sets of variables (adjusted for the main effects of Monte-Carlo repetitions). We now summarize the key results of this ANOVA, which generalises the intuitions provided by the simulation depicted in Fig. 4.

We tested for the significance of main effects and multiway interactions on estimation accuracy (MSE) and confidence bias (MCB) on all three sets of variables (input, states and evolution parameters). The details of this analysis are reported in the Supplementary materials. Here, we summarize the results, focusing on interesting interactions.

The Monte-Carlo simulations suggest that modelling neural noise (using either sDCM or dDCM augmented with temporal basis functions) seems to improve overall estimation accuracy. Note that using basis functions with stochastic DCM reduces estimation accuracy (because random fluctuations are effectively modelled twice). However, the advantage of modelling noise depends on the properties of the fMRI data and the underlying neural dynamics; e.g., *SNR* and the presence of nonlinearities in the neural evolution function:

– Neural state estimation improves overall, when neural noise is modelled (sDCM or augmented dDCM), particularly in the presence of nonlinearities.
– Temporal basis functions provide poorer estimates of random fluctuations for both sDCM and dDCM. However, sDCM is found to be more conservative (less over confident) than dDCM, particularly at low SNR.
– Temporal basis functions do not improve the estimation of evolution parameters for dDCM, which performs worse than sDCM in the presence of neural noise. However, sDCM is more capricious (more over confident) than dDCM in the absence of nonlinearities.

Note that, on average, the presence of nonlinearities in the neural evolution function aggravates the over confidence of dDCM but not of sDCM — this aspect of dDCM is particularly apparent in the (realistic) low *SNR* case and in the presence of neural noise.

### Monte-Carlo simulations: assessing model comparison

Above, we established that estimation accuracy can, in some instances, be improved by including state noise in the generative model. However, this is not entirely predictive of the accuracy of Bayesian model comparison, which reduces all the information in the data to a qualitative decision problem. Here, we asses Bayesian model comparison directly: that is we ask whether modelling state noise can help to discriminate between models that differ in terms of their connectivity structure. More precisely, we examine the problem of identifying *mediated influences* in the network, a notoriously difficult problem (see Valdés-Sosa et al., 2011).

**Fig. 11:**
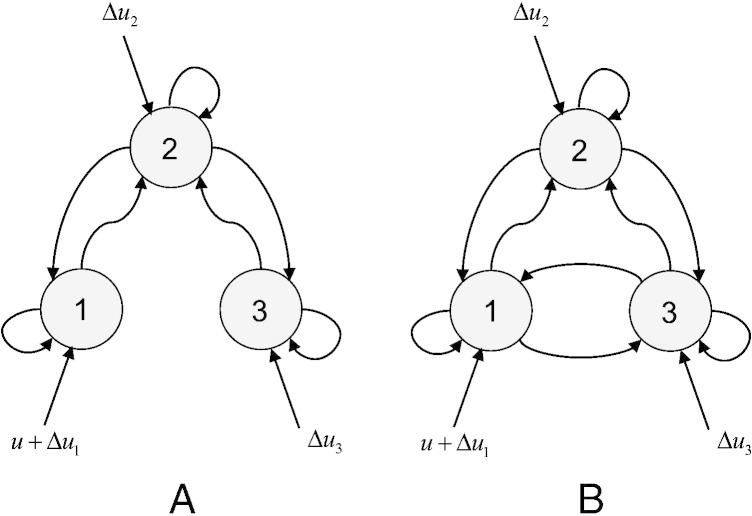
Discriminating between direct and mediated effects: canonical scenarios. This figure summarizes the two canonical models considered when discriminating between direct and mediated influences (A — mediated influence, B — mediated and direct influence). Note that node 1 is always driven by a known experimental stimulation *u*, but that all nodes can be perturbed by independent input fluctuations *Δu* (see main text).

Here, we focus on the ‘canonical’ problem of identifying mediation in the presence of reciprocal connections, as is typical for neuronal circuits (Kötter & Stephan 2003). Our canonical scenario is summarised by Fig. 11:

• In scenario A, the causal effect of node 1 on node 3 is *mediated by* node 2.
• In scenario B, all nodes are (reciprocally) connected to each other; that is, there is a *direct* effect of node 1 onto node 3 in addition to the mediated effect.

Both scenarios induce statistical dependencies between nodes 1 and 3. However, effective connectivity analyses should, ideally, identify direct reciprocal influences between nodes 1 and 3 in case A only. We therefore asked whether modelling state noise increases the discriminability of these two models, using the log-Bayes factor *LBF*_*AB*_ (Daunizeau et al., 2011):

(20) $$LBF_{\mathrm{AB}}=\log\frac{p\left(y|A,m\right)}{p\left(y|B,m\right)}\text{.}$$

Here *m* represents a stochastic or a deterministic DCM. *LBF*_*AB*_ is a signed discrimination power measure: for *LBF*_*AB*_ ≫ 0, there is strong evidence in favour of a mediated influence; conversely, *LBF*_*AB*_ ≪ 0 represents strong evidence in favour of a direct influence. We ran a 2 × 2 factorial Monte-Carlo simulation series to compare the respective discrimination power of stochastic and deterministic DCM, with the following two factors:

• *Network*: fMRI data were simulated either under model A (mediated influence) or under model B (direct and mediated influence).
• *Δu*: The simulated system was either driven by an exogenous input *u* or by the same input plus some neural fluctuations *Δu*.

For each combination of these factors, we ran 24 simulations, producing 96 simulated sessions in total. Each of these sessions was then subject to model inversion, using a 2 × 2 factorial model set with the following factors:

• *Network*: the generative model assumed a network structure of type A or type B.
• *DCM type*: the generative model assumed the presence or absence of state noise *ϖ* (i.e., stochastic or deterministic DCM).

Note that, following the results of the previous section, we did not use temporal basis functions (because they decreased accuracy performance on average) and we fixed the signal-to-noise ratio to 1 dB (low SNR level). All other simulation parameters were identical to the previous simulation series. We used the network factor to compute, for each data set and DCM type, the log-Bayes factor *LBF*_*AB*_. Discriminability (*LBF*_*AB*_) was analysed using a 2 × 2 × 2 ANOVA (within Monte-Carlo repetitions; N = 24).

**Fig. 12:**
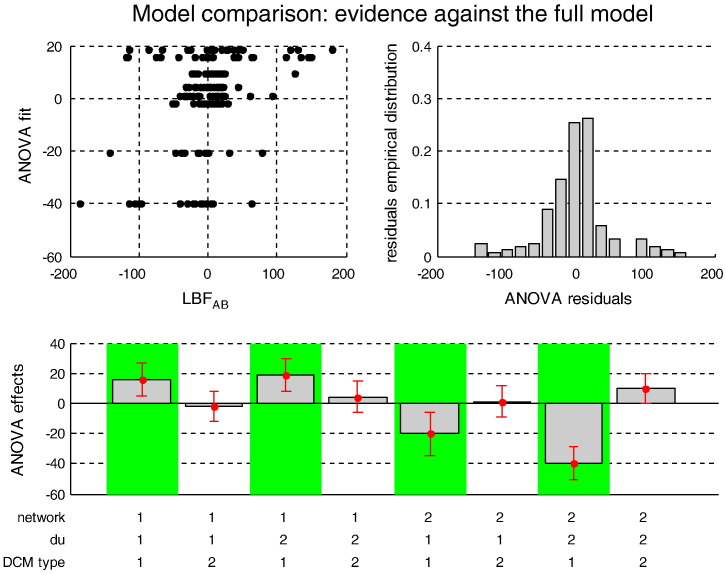
Model comparison results: LBF_AB_. This figure summarizes the results of the Monte-Carlo simulations, in terms of the estimated LBF_AB_ as a function of the three factors of the simulation design. Upper-left: the best fit of the ANOVA (y-axis) is plotted against the actual observed LBF_AB_ (x-axis) for each individual simulation. Upper right: the empirical histogram of the ANOVA residuals. Lower half: the Monte-Carlo estimate of the mean effect on LBF_AB_ for each of the 8 cells of the factorial design: *network* = 1: data simulated under model A, *network* = 2: data simulated under model B (see Fig. 11); *du* = 1: no input fluctuation, *du* = 2: input fluctuations; *DCM type* = 1: sDCM (these cells are highlighted using the green shaded areas), *DCM type* = 2: dDCM.

Fig. 12 summarizes the results of this ANOVA, in terms of the estimated effects of each of the 8 conditions on *LBF*_*AB*_ (adjusted for the main effects of Monte-Carlo repetitions). Again, we refer the interested reader to the Supplementary materials for details of the statistical analysis. Below, we summarize the results, focusing on non-trivial interactions.

Overall, the Monte-Carlo simulations show that, on average, dDCM cannot discriminate between networks A and B, irrespective of the presence of neural noise. In contradistinction, sDCM appears to exploit neural noise to distil discriminative evidence from the fMRI data. This is because, on average, neural noise de-correlates the trajectories of hidden neural states. In the final section, we turn to an application of stochastic DCM, in which fluctuations in neuronal states are not under experimental control.

## Application to an fMRI study of an epileptic crisis

In this section, we compare stochastic and deterministic DCM in the context of paradigm-free fMRI experiments. We conducted an sDCM analysis of data from an epileptic patient, who experienced an absence seizure in the fMRI scanner. These data were part of a previous neuroimaging study of idiopathic generalized epilepsy (Hamandi et al., 2006; Vaudano et al., 2009), whose acquisition protocol and data pre-processing are briefly summarized here.

Ten-channel EEG (10–20 system) was recorded using MR-compatible equipment, along with bipolar electrocardiogram. After filtering and artifact correction (Krakow et al., 2000), the onsets and offsets of generalized spike and wave (GSW) activity were identified by two experts (see Vaudano et al., 2009 for details). Four hundred and four T2*-weighted single-shot gradient echo echo-planar images (TE = 40 ms, TR = 3 s, 21 interleaved axial slices of 5 mm thickness, FOV = 24 × 24 cm^2^, 64 × 64 matrix) were acquired over a twenty minute session on a 1.5 Tesla MRI scanner (Horizon EchoSpeed, General Electric). The patient was asked to rest with his eyes closed and to keep still. FMRI data were pre-processed using SPM8 (http://www.fil.ion.ucl.ac.uk/spm/). EPI time series were realigned and spatially smoothed with an 8 mm FWHM isotropic Gaussian kernel and normalized. A general linear model (GLM) was constructed to assess the presence of regional GSW-related BOLD changes. GSW activity was modelled as two blocks, beginning at GSW onset and terminating at their offset. The GSW block regressor was then convolved with the canonical hemodynamic response function (plus temporal and dispersion derivatives) before inclusion in the GLM. Both motion-related effects (both head and eye movements) and cardiac confounds (see Liston et al., 2006) were included as regressors. In addition to the above confounding factors, scan-nulling regressors (modeling inter-scan motion events larger than 2 mm) were included in the GLM (Lemieux et al., 2007). In total, the GLM design matrix contained ninety three regressors, three of which were of interest.

**Fig. 13:**
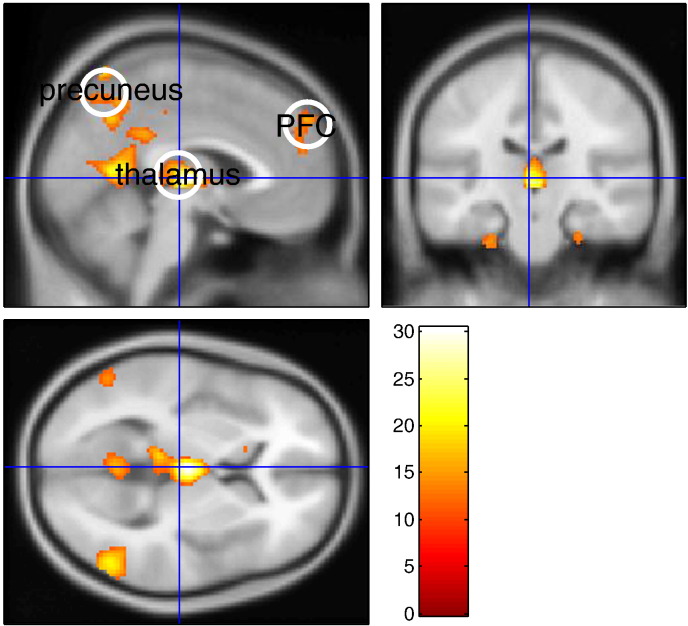
Absence seizure analysis: regions of interest. This figure summarizes the standard SPM activation results of a single case study of an epileptic (petit mal) absence seizure. Significant (whole brain FWE-corrected) positive and negative GSW-related BOLD responses were identified using an F-contrast on the GSW regressors. The colour bar indicates the range of displayed F values.

Fig. 13 shows the significant positive and negative GSW-related BOLD responses that were identified by means of an F-contrast on the GSW regressors. The resulting SPM was thresholded at p < 0.05 (FWE whole-brain corrected) to define three regions of interest (ROIs), which were involved in the initiation and termination of GSW discharges: thalamus, prefrontal cortex (PFC) and precuneus. A summary time series was derived for each ROI by computing the first eigenvariate of all suprathreshold voxel time series within a 10 mm of the ROI centres, which are given in Table 2 below. The time series were corrected for all confounding effects included in the GLM analysis.

We specified a series of DCMs for this 3-region network based on the following considerations: first, we wanted to address the qualitative nature of GSW activity in this patient. More precisely, we wanted to know whether GSW activity is best described by a sudden increase in the external input to the network, or by an abrupt change in connectivity strength. This distinction directly maps to modelling the GSW input either as a driving (C matrix of Eq. (9)) or modulatory (B matrix of Eq. (9)) input. For each of these two explanations, we considered four models, which differ in (i) C-family: where GSW input enters the network (c.f. Vaudano et al., 2009) and (ii) B-family: which connections are specifically modulated within the cortico-thalamic loop. We also included a “null” scenario, in which GSW input had no effect. Note that all DCMs have full reciprocal connectivity (A matrix in Eq. (9)).

**Fig. 14:**
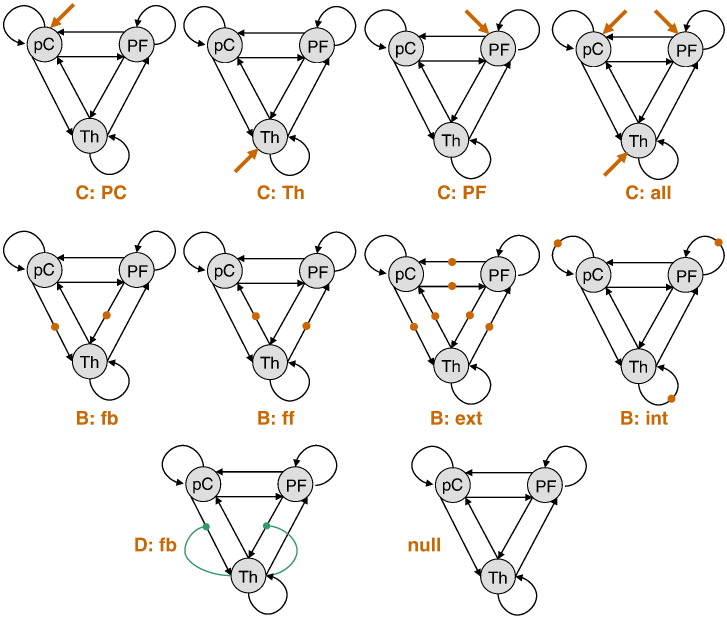
Absence seizure analysis: DCM comparison set. This figure summarizes the factorial model comparison set (models space), which comprises three factors (nature of GSW transient change, presence of nonlinear thalamic gating and stochastic versus deterministic). Note that all networks are fully connected (state–state couplings). The first two rows depict the two families of the first factor; i.e. whether GSW input drives the network (models C: the orange arrows show where GSW enters the network) or GSW input modulates connections within the network (models B: the orange dots show which connections are allowed to transiently change during GSW activity). In addition to these two families, the null model (lower-right graph) assumes that there is no change associated with GSW activity. The second factor is whether or not thalamic afferent connections are gated by activity in the thalamus (model D: the green arrows indicate nonlinear thalamic gating). Each of the other 9 models thus has two variants, with and without nonlinear thalamic gating. Lastly, each of these 9 × 2 = 18 models can be inverted under either a stochastic or a deterministic DCM. In total, 36 models comprise the models space.

This model space was motivated by the standing debate regarding the putative role of the thalamus and the cortex as GSW generators (see, for example, Steriade and Contreras, 1998). In addition, the literature on absence epilepsy also highlights GABAergic influences from the reticular thalamic nucleus on other (dorsal) thalamic nuclei; these determine the excitability of thalamic cells and thus the influence of descending corticothalamic inputs on thalamic cells (Steriade, 2005; Huguenard and McCormick, 2007; Schofield et al., 2009). We therefore added a second factor to our model space; namely, whether or not the thalamus regulates its own sensitivity to cortical inputs. Each of the 9 models above thus had two variants — with and without nonlinear thalamic gating (D matrix in Eq. (9)). The ensuing model comparison set is summarized in Fig. 14.

Finally, we considered both the deterministic and the stochastic variants of each of these 18 DCMs, which constitute the last factor in our 9 × 2 × 2 factorial model comparison. In total, 36 models were considered in this study. Note that only models belonging to the C-family can elicit non-zero activity in the network under a deterministic DCM framework. This means that all dDCM models belonging to the B-family are effectively null models. All models were equipped with priors that were identical to those used in the simulations above, except that we relaxed the prior variance on hemodynamic parameters (*Ψ* = 10^− 2^). This is because it has been shown that hemodynamic responses in epilepsy can deviate from their canonical form (Grouillet et al., 2010).

**Fig. 15:**
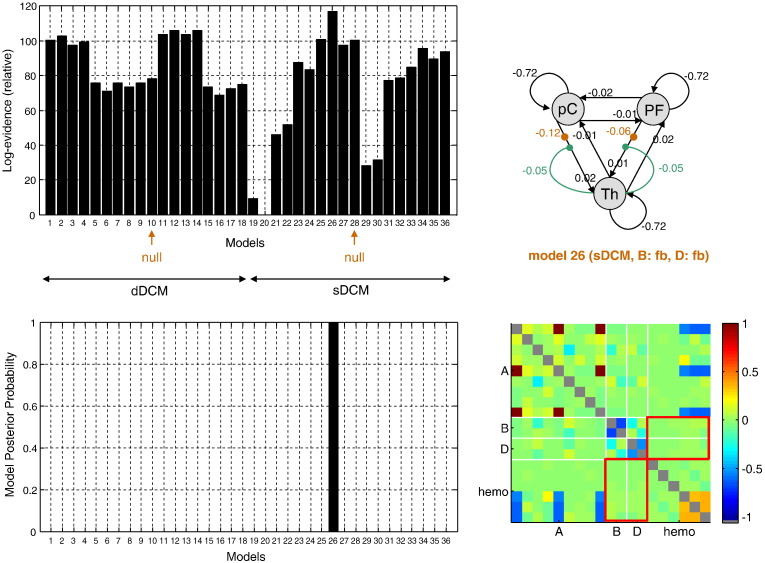
Absence seizure analysis: model comparison results. This figure summarizes the results of the DCM analysis of fMRI data. Upper-left: free energy (relative to model 20) of each model included in the comparison set (c.f. Fig. 14). The first half of the models are deterministic, whereas the second half are stochastic. For both DCM variants, the null model without thalamic gating is indicated by an orange arrow. Lower-left: induced model posterior probabilities. Upper-right: This graph summarizes the estimated structure of the winning model; i.e. model 26 (*sDCM*, *B*: *fb*, *D*: *fb*), which assumes a transient change in the thalamic afferent connections during the seizure, as well as nonlinear thalamic gating. Lower-left: posterior correlation matrix of the evolution parameters under model 26. The matrix is partitioned into sections that correspond to the A, B and D matrices of the neural evolution function (c.f. Eq. (9)), as well hemodynamic parameters (c.f. Eq. (10)).

The results of Bayesian model comparison are summarized in Fig. 15, in terms of (the free energy bound on) log model evidence (Eq. (14)). The model comparison results show that the most plausible model depends on whether the stochastic or the deterministic variant of DCM is used. Overall, however, the stochastic DCM with nonlinear gating and transient modulation of thalamic afferent connections (model 26: *sDCM*, *B: fb*, *D: fb*) is the most likely explanation for GSW-related activity. Note that this model is the only model that is more probable than the null stochastic DCM, which assumes that there is no transient change in the network during the GSW crisis.

In addition, we inspected the moments of the posterior distribution *q*(*θ*) of the evolution parameters. Note that close-to-zero non-diagonal elements in the posterior correlation matrix indicate that the corresponding parameters are uniquely identifiable. In brief:

– The average level of connectivity within the network “at rest” (A matrix of Eq. (9)) seems to be rather low, when compared to the self-inhibitory time constant. However, these parameters are only partly identifiable due to correlations with hemodynamic parameters (compare the posterior correlation matrix in Fig. 15).
– The nonlinear gating and transient modulation of thalamic feedback connections are almost perfectly identifiable and not affected by hemodynamic parameters (see the red rectangles in the posterior correlation matrix of Fig. 15). Their sign shows that (i) in normal conditions, the thalamus acts as a self-stabilizing de-amplifying device and (ii) a GSW crisis is associated with a transient thalamic disinhibition through the cortico-thalamic loop.

In conclusion, we have shown that GSW activity is better explained by a transient change in network connectivity, rather than by a sudden increase in exogenous input to the system. This distinction is important because the former scenario explains the occurrence of a GSW seizure in terms of network properties, rather than a local change in excitatory activity. The key point here is that – in our analyses – the transient change in the network activity (as measured using BOLD fMRI) could be explained without changing the statistical properties of the input to the system; this would have been impossible without including neural noise in the generative model.

## Discussion

In this work, we have assessed stochastic DCM in relation to deterministic variants. First, we reviewed the theoretical properties of stochastic dynamical systems, in terms of the impact that state noise can have on brain network dynamics. In brief, stochastic effects can be profound when there are nonlinear interactions among hidden states that render systems non-quasi deterministic. We then reported a comprehensive evaluation of the respective system identification ability of stochastic and (suitably extended) deterministic DCMs. In summary, our Monte-Carlo simulations showed that both variants of DCM can, in principle, account for (unknown) neural fluctuations or noise. However, their relative performance in terms of network identification depends upon signal-to-noise ratios and nonlinearities in the neural evolution function. For example, in contradistinction to stochastic DCM, the over confidence of deterministic DCM is (on average) aggravated by nonlinearities in the neural evolution function. This is consistent with the theoretical analysis of the first section — in the sense that the increase in the dynamical repertoire of such non quasi-deterministic systems (induced by the presence of neural noise) seems to confound deterministic DCM identification schemes. This is important, because overconfidence can impact on both parameter estimation and model selection (c.f. Eq. (14): the free energy is an explicit function of the approximate posterior covariance). In addition, we have shown that stochastic DCM can exploit the presence of neural noise to discriminate between mediated and direct influences within networks. This is important, because this means that neural noise may actually improve the identification of network structure. Finally, our empirical application demonstrated the sorts of inference that can be drawn using stochastic DCM of data, whose variance is not (or poorly) controlled by experimental manipulations.

Generally speaking, model identification in the context of stochastic DCM for fMRI data involves (i) selecting a model, (ii) estimating the associated parameters and (iii) recovering hidden neuronal states. Our results demonstrate that the accuracy of model identification depends upon the nature of the model implicit in its priors. We will now discuss the non trivial issue of choosing priors *p*(*ϖ*|*m*) on state noise. Recall that we considered deterministic and stochastic DCM, both with and without temporal basis functions. This factorial model space is defined in terms of priors over temporal correlations among neural fluctuations. This is summarized in Table 3.

Although stochastic and deterministic DCMs also differ in terms of model inversion (see the comment on hemodynamics below), we believe that the main determinant of their relative performance is prior assumptions about state noise. This is because we found that the use of temporal basis functions had a significant impact on estimation accuracy, irrespective of whether the models were stochastic or deterministic. Having said this, we have shown that using basis functions is almost always detrimental to stochastic DCM. This is probably due to a redundant modelling of state noise, which compromises system identifiability. In short, the relative performance of deterministic and stochastic DCM can be understood as a (non-trivial) consequence of prior assumptions about state noise. This is because these priors control the relative identifiability of the two components of network dynamics, namely activity propagation (through network connectivity) and driving forces that act locally on the system. This is the reason why one may want to estimate the auto-correlation or smoothness of state noise by including it as a parameter of the generative model. This can be done through inversion schemes such as dynamic expectation maximization (DEM) (Friston et al., 2008) or generalised filtering (Friston et al., 2010), which rely upon generalized coordinates (Eqs. (3) and (4)) to relax the restrictive (Markovian) assumptions of classical state space models (Valdés-Sosa et al., 2011). Recall that a Taylor expansion in time provides a linear mapping from instantaneous generalized motion to entire time series:

(23) $$\begin{array}{c} \varpi \left(t+\Delta t\right)=\varpi \left(t\right)+\Delta t\;\dot{\varpi }\left(t\right)+\frac{1}{2}\Delta t^{2}\ddot{\varpi }\left(t\right)+\ldots \\ =\left[\begin{array}{ccccc} 1 & \Delta t & \frac{1}{2!}\Delta t^{2} & \ldots & \frac{1}{d!}\Delta t^{d} \end{array}\right]\tilde{\varpi }\left(t\right) \end{array}$$

where the time lag *Δt* is arbitrary and *d* is the embedding dimension (the order of generalized motion). From Eq. (23), it is easy to see how any non-zero lagged-covariance (between, e.g., *ϖ*(*t*) and *ϖ*(*t* + *Δt*)) can be rewritten in the form of non-zero extra-diagonal terms in the instantaneous covariance matrix of generalized coordinates (see Equation 52 in Friston et al., 2008). Thus, expressing the generative model in generalized coordinates of motion (Eqs. (3) and (4)) allows us to model any lagged dependencies on state noise and thus estimate the unknown smoothness during model inversion. We will evaluate the utility of generalized hidden states and hierarchical priors on state noise in future work.

It is worth highlighting that, in addition to signal-to-noise ratio, the presence of nonlinearities and the presence of neural noise, there are other factors that determine the relative performance of DCM variants. For example, both the fMRI sampling rate and overall session duration are likely to influence estimation accuracy. The impact of, for example, session duration on dDCM should be similar to SNR, since it effectively determines how much measurement noise can be averaged out across repetitions of experimental inputs. However, its effect on sDCM, or its interaction with other factors (such as the presence of nonlinearities) is less trivial. This is because the number of sDCM unknown variables scales with the session duration (cf. hidden states). Furthermore, experimental design (e.g., the number of controlled inputs to the network) can have a strong impact. In the limit, a design that is maximally powerful for addressing a particular inference problem (e.g., comparing two network structures), will eliminate differences in the relative performance of DCM variants (cf. Daunizeau et al., 2011). Last but not least, the complexity and dimension of the network can have a strong influence on the overall performance of network identification (Smith et al., 2011). We discuss issues related to the computational cost below, but we expect this last factor to be critical. This is because the identification of high-dimensional networks is likely to be very demanding in terms of experimental design and data quality. This means there probably is an upper limit to the dimension of identifiable networks.

One of the main (and unexpected) difficulties of stochastic DCM is inherent in haemodynamic modelling, which turned out to be more problematic than for dDCM. In brief, the dynamical structure of the hemodynamic model (Eqs. (10) and (11)) means that the causal impact of neural states on observed BOLD signals is delayed: a particular fMRI time sample *y*(*t*) contains information about past neural states *x*(*t* − *Δt*), where the delay *Δt* is about 5 s. Equivalently, current neural states *x*(*t*) only impact on the third-order motion $\overset{\cdots }{y}\left(t\right)$ of BOLD signals. Under high levels of hemodynamic state noise, such an indirect mapping might cause instability during the inversion, i.e. limit the ability of sDCM to recover the neural state dynamics and the network structure. This problem is partly finessed by using very precise priors on the fluctuations of haemodynamic states, which resolves conditional dependencies between (mediated) neuronal and (direct) haemodynamic contribution to the observed BOLD signal. In the absence of generalised states, one has to ensure that the posterior density on hidden states at time *t* is conditioned on data up to time *t* + *Δt* (see the Appendix A for details). We found that a lag *Δt* = 6 s was sufficient to obtain efficient and robust estimates of neural states, but we had to increase it up to *Δt* = 16 s in order to achieve accurate estimation of the precision of state noise *λ*_*ϖ*_ (results not shown). As with the embedding dimension for generalized coordinates (Eq. (23)), increasing the lag has a computational cost: overall, sDCM is about thirty times slower than dDCM. However, this is not specific to stochastic DCM: including neural noise in the generative model (using either sDCM or augmented dDCM) will inevitably inflate the computational cost of model inversion. More precisely, in either case, if *n* is the number of regions, the computational cost is *O*(*n*^3^) We refer to Daunizeau et al. (2009) for a more exhaustive analysis of the scalability of stochastic DCM.

We will now discuss the analysis of the empirical data, and consider the results in terms of validation of stochastic DCM and their neurobiological implications. In terms of model comparison, one of the more complex models was selected (*sDCM*; *B*: *fb*, *D*: *fb*). One might wonder whether this reflects some form of over-fitting. However, a careful inspection of the model evidences (Fig. 15) makes this explanation unlikely. For example, the next to best sDCM is the (simple) null model. This illustrates the ability of the free energy approximation to the log-evidence (see Eq. (15)) to measure how much added accuracy is needed to compensate for the extra complexity of a model.

**Fig. 16:**
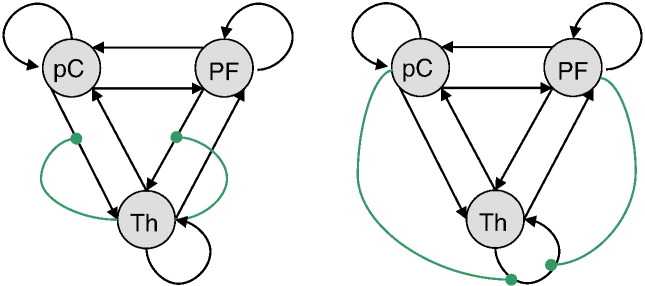
Ambiguity of nonlinear thalamic gating. This figure shows two possible connectivity structures for nonlinear thalamic gating effects (green arrows). Left: the interpretation that was implicitly used throughout the manuscript. Right: a qualitatively different interpretation of the nonlinear gating effects (but statistically equivalent in that this graphical representation rests upon the same state equations).

Finally, the reader might realize (and find unfortunate) that the nonlinear thalamic gating (model *D: fb*) can be interpreted in at least two qualitatively different ways (see Fig. 16). However, it seems reasonable to speculate about thalamic gating in the generation of GSW absence seizures. Even though this case study does not capture the complexity of GSW transition mechanisms, the presence of nonlinear gating effects has important dynamical implications: for example, thalamic gating acts as a de-amplifier of cortical activity (*D* < 0, see Fig. 15), and the system is multistable. These dynamical properties are consistent with the recent literature concerning the relation of both the precuneus and the PFC to reduced vigilance and altered states of consciousness (see, e.g., Laureys et al., 2004; Faymonville et al., 2006). Furthermore, these properties are also consistent with a phasic thalamic gating of cortical projections from the midbrain during attentional shifts (that effectively suppress cortically driven action selection: Floresco and Grace, 2003; Ding et al., 2010). We will report a generalization of these results, at the group level, in forthcoming publications.

## Figures and Tables

**Table 1:** Hemodynamic parameters.

Constant	Physical meaning	Value	Physical unit
*κ*_*s*_	Vasodilatory signal decay rate	0.65	s^− 1^
*κ*_*f*_	Vasodilatory signal feedback rate	0.41	s^− 1^
*τ*_0_	Mean transit time	2	s
*α*	Vessel stiffness	0.32	–
*E*_0_	Oxygen extraction fraction at rest	0.34	–
*V*_0_	Venous volume fraction at rest	4	–
*ν*_0_	Frequency offset	40.3	s^− 1^
*TE*	EPI echo time	0.04	s
*r*_0_	Intravascular relaxation rate	25	s^− 1^
*ε*_0_	ratio of intra- and extra-vascular signals	1	–

**Table 2:** Empirical data DCM analysis: ROIs centres (MNI coordinate system) and number of suprathreshold voxels.

ROI	x	y	z	# of voxels
Precuneus	− 6	− 58	50	100
Thalamus	− 2	− 18	4	218
PFC	− 18	46	46	167

**Table 3:** Prior covariance over state noise.

	Without input basis set	With input basis set
dDCM	No fluctuations	Serially correlated
sDCM	Markovian (uncorrelated)	Mixture of uncorrelated and serially correlated

## Appendix A

This section deals with the derivation of the lagged states' posterior density *p*(*x*_*t* − *k*_|*y*_1 : *t*_), where *k* is the lag. This effectively means hidden states estimates *x*_*t*_ is conditioned on data up to time *t* + *k*. This is useful when the contribution of some states to the data only appears away in future time, as is the case in DCM for fMRI. We are concerned with Markovian state-space models, which are defined in terms of a transition density *p*(*x*_*t*_|*x*_*t* − 1_) and a likelihood function *p*(*y*_*t*_|*x*_*t*_). Note that the following scheme generalizes to hierarchical models with parameterized likelihood and transition densities. This is because, under the usual mean-field assumption of variational Bayesian approaches, iterative approximate marginalization follows (Daunizeau et al., 2009):

A0 $$\begin{array}{c} p\left(y_{t}|x_{t}\right)\overset{VB}{\leftarrow }\exp\langle \log p\left(y_{t}|x_{t},\varphi \right)\rangle \\ p\left(x_{t+1}|x_{t}\right)\overset{VB}{\leftarrow }\exp\langle \log p\left(y_{t}|x_{t},\theta \right)\rangle \end{array}$$

where *θ* (resp. *φ*) are observation parameters (including, e.g., second-order precision hyperparameters), and the expectation is taken under their current approximate posterior density.

Let us first consider the derivation of the joint density *p*(*x*_*t* − *k*_, *x*_*t* − *k* + 1_, …, *x*_*t*_|*y*_1 : *t*_):

A1 $$\begin{array}{l} p\left(x_{t-k},\ldots ,x_{t}|y_{1:t}\right)=p\left(y_{t}|x_{t-k},\ldots ,x_{t}\right)p\left(x_{t-k},\ldots ,x_{t}|y_{1:t-1}\right)\\ =p\left(y_{t}|x_{t}\right)p\left(x_{t-k},\ldots ,x_{t}|y_{1:t-1}\right)\\ =p\left(y_{t}|x_{t}\right)\int p\left(x_{t-k-1},\ldots ,x_{t}|y_{1:t-1}\right)dx_{t-k-1}\\ =p\left(y_{t}|x_{t}\right)\int p\left(x_{t}|x_{t-k-1},\ldots ,x_{t-1}\right)p\left(x_{t-k-1},\ldots ,x_{t-1}|y_{1:t-1}\right)dx_{t-k-1}\\ =p\left(y_{t}|x_{t}\right)\int p\left(x_{t}|x_{t-1}\right)p\left(x_{t-k-1},\ldots ,x_{t-1}|y_{1:t-1}\right)dx_{t-k-1}\\ =p\left(y_{t}|x_{t}\right)p\left(x_{t}|x_{t-1}\right)\int p\left(x_{t-k-1},\ldots ,x_{t-1}|y_{1:t-1}\right)dx_{t-k-1} \end{array}$$

where the fourth and the fifth lines result from conditional independence assumptions induced by the Markovian structure of the generative model.

Eq. (A1) tells us how to transform the ‘previous’ joint density *p*(*x*_*t* − *k* − 1_, …, *x*_*t* − 1_|*y*_1 : *t* − 1_) into the ‘current’ joint *p*(*x*_*t* − *k*_, …, *x*_*t*_|*y*_1 : *t*_). This is the basis for the lagged forward pass we are looking for, because marginalizing this joint density over the states *x*_*t* − *k* + 1_, …, *x*_*t*_ yields the lagged posterior density *p*(*x*_*t* − *k*_|*y*_1 : *t*_):

A2 $$\begin{array}{l} p\left(x_{t-k}|y_{1:t}\right)=\int p\left(x_{t-k},\ldots ,x_{t}|y_{1:t}\right)dx_{t-k+1}\cdots dx_{t}\\ =\int p\left(y_{t}|x_{t}\right)p\left(x_{t}|x_{t-1}\right)\left[\int p\left(x_{t-k-1},\ldots ,x_{t-1}|y_{1:t-1}\right)dx_{t-k-1}\right]dx_{t-k+1}\cdots dx_{t} \end{array}$$

Let us now focus on the nonlinear Gaussian case, whereby the transition density and the likelihood are given by (c.f. Eqs. (1) and (2) in the main text):

A3 $$\left\{ \begin{array}{l} p\left(x_{t}|x_{t-1}\right)=N\left(f\left(x_{t-1}\right),Q_{x}\right)\\ p\left(y_{t}|x_{t}\right)=N\left(g\left(x_{t}\right),Q_{y}\right) \end{array}\right.$$

where *f* (resp. *g*) is the evolution (resp. observation) function, and *Q*_*x*_ (resp. *Q*_*y*_) is the second-order moment of the transition density (resp. likelihood function).

Furthermore, let us note $X_{t}={\left[\begin{array}{ccc} x_{t-k}{}^{T} & \cdots & x_{t}{}^{T} \end{array}\right]}^{T}$ and define the following four operators:

A4 $$\begin{array}{l} C=\left[\begin{array}{cc} 0_{n\times n\left(k-1\right)} & I_{n} \end{array}\right]\Rightarrow CX_{t}=x_{t}\\ D=\left[\begin{array}{ccc} 0_{n\times n\left(k-2\right)} & I_{n} & 0_{n\times n} \end{array}\right]\Rightarrow DX_{t}=x_{t-1}\\ E=\left[\begin{array}{cc} I_{n\left(k-1\right)} & 0_{n\left(k-1\right)\times n} \end{array}\right]\Rightarrow EX_{t}={\left[\begin{array}{ccc} x_{t-k}{}^{T} & \cdots & x_{t-1}{}^{T} \end{array}\right]}^{T}\\ \overset{⌣}{E}=\left[\begin{array}{cc} 0_{n\left(k-1\right)\times n} & I_{n\left(k-1\right)} \end{array}\right]\Rightarrow \overset{⌣}{E}X_{t-1}=EX_{t} \end{array}$$

where *n* is the dimension of the state space.

We are now in a position to derive the lagged update rule.

Let us assume that we have found a Gaussian approximation to the previous joint posterior density *p*(*X*_*t* − 1_|*y*_1 : *t* − 1_) ≈ *N*(*m*_*t* − 1_, Σ_*t* − 1_). Using Eq. (A4), this implies that:

A5 $$\begin{array}{l} \int p\left(x_{t-k-1},\ldots ,x_{t-1}|y_{1:t-1}\right)dx_{t-k-1}=p\left(\overset{⌣}{E}X_{t-1}|y_{1:t-1}\right)\\ =N\left(\overset{⌣}{E}m_{t-1},\overset{⌣}{E}\Sigma_{t-1}{\overset{⌣}{E}}^{T}\right)\\ =p\left(EX_{t}|y_{1:t-1}\right) \end{array}$$

where the first and last lines come from the definition of the linear operators $\overset{⌣}{E}$ and *E* (see Eq. (A4)). Inserting Eqs. (A3) and (A5) into Eq. (A1) gives the current joint density *p*(*X*_*t*_|*y*_1 : *t*_):

A6 $$\begin{array}{l} \log p\left(X_{t}|y_{1:t}\right)=c_{0}+\log p\left(y_{t}|x_{t}\right)+\log p\left(x_{t}|x_{t-1}\right)+\log p\left(\overset{⌣}{E}X_{t-1}|y_{1:t-1}\right)\\ =c_{0}-\frac{1}{2}{\left(y_{t}-g\left(x_{t}\right)\right)}^{T}Q_{y}{}^{-1}\left(y_{t}-g\left(x_{t}\right)\right)\\ -\frac{1}{2}{\left(x_{t}-f\left(x_{t-1}\right)\right)}^{T}Q_{x}{}^{-1}\left(x_{t}-f\left(x_{t-1}\right)\right)\\ -\frac{1}{2}{\left(\overset{⌣}{E}m_{t-1}-EX_{t}\right)}^{T}{\left[\overset{⌣}{E}\Sigma_{t-1}{\overset{⌣}{E}}^{T}\right]}^{-1}\left(\overset{⌣}{E}m_{t-1}-EX_{t}\right) \end{array}$$

where *c*_0_ is a normalization constant and the last line follows directly from Eq. (A5). Eq. (A6) can now be turned into a quadratic form in *X*_*t*_ through a Taylor expansion of both evolution and observation mappings around a given point *Γ*_*t*_:

A7 $$\left\{ \begin{array}{l} g\left(x_{t}\right)=g\left(CX_{t}\right)\approx g\left(C\Gamma_{t}\right)+GC\left(X_{t}-\Gamma_{t}\right)\\ f\left(x_{t-1}\right)=f\left(DX_{t}\right)\approx f\left(D\Gamma_{t}\right)+FD\left(X_{t}-\Gamma_{t}\right) \end{array}\right.$$

where the gradients *G* ≜ ∂ *g*/∂ *x* (resp. *F* ≜ ∂ *f*/∂ *x*) are evaluated at the expansion points *CΓ*_*t*_ (resp. *DΓ*_*t*_). Inserting Eq. (A7) into Eq. (A6) yields:

A8 $$\begin{array}{l} \begin{array}{c} \log p\left(X_{t}|y_{1:t}\right)\approx c_{0}+-\frac{1}{2}{\left(y_{t}+\varepsilon_{t}-GCX_{t}\right)}^{T}Q_{y}{}^{-1}\left(y_{t}+\varepsilon_{t}-GCX_{t}\right)\\ -\frac{1}{2}{\left(\eta_{t}-\left(FD-C\right)X_{t}\right)}^{T}Q_{x}{}^{-1}\left(\eta_{t}-\left(FD-C\right)X_{t}\right)\\ -\frac{1}{2}{\left(\overset{⌣}{E}m_{t-1}-EX_{t}\right)}^{T}{\left[\overset{⌣}{E}\Sigma_{t-1}{\overset{⌣}{E}}^{T}\right]}^{-1}\left(\overset{⌣}{E}m_{t-1}-EX_{t}\right) \end{array}\\ \begin{array}{c} \varepsilon_{t}≜GC\Gamma_{t}-g\left(C\Gamma_{t}\right)\\ \eta_{t}≜FD\Gamma_{t}-f\left(D\Gamma_{t}\right) \end{array} \end{array}$$

where *ε*_*t*_ and *η*_*t*_ play the role of (Taylor) expansion error signals (they are identically zero if the evolution and observation functions are linear). Eq. (A8) can be rewritten as an explicit quadratic function of the lagged states *X*_*t*_ of the form $-\frac{1}{2}{\left(m_{t}-X_{t}\right)}^{T}\Sigma_{t}{}^{-1}\left(m_{t}-X_{t}\right)$, where, by definition:

A9 $$\begin{array}{l} m_{t}:{\frac{\partial }{\partial X_{t}}\log p\left(X_{t}|y_{1:t}\right)|}_{m_{t}}=0\\ \Rightarrow m_{t}=\Sigma_{t}\left(C^{T}G^{T}Q_{y}{}^{-1}\left(y_{t}+\varepsilon_{t}\right)+{\left(FD-C\right)}^{T}Q_{x}{}^{-1}\eta_{t}+E^{T}{\left[\overset{⌣}{E}\Sigma_{t-1}{\overset{⌣}{E}}^{T}\right]}^{-1}\overset{⌣}{E}m_{t-1}\right)\\ \Sigma_{t}≜-{\left[\frac{\partial^{2}}{\partial X_{t}{}^{2}}\log p\left(X_{t}|y_{1:t}\right)\right]}^{-1}\\ ={\left[C^{T}G^{T}Q_{y}{}^{-1}GC+{\left(FD-C\right)}^{T}Q_{x}{}^{-1}\left(FD-C\right)+E^{T}{\left[\overset{⌣}{E}\Sigma_{t-1}{\overset{⌣}{E}}^{T}\right]}^{-1}E\right]}^{-1} \end{array}$$

Eq. (A9) gives the update rule for the two first-order moments of the current joint posterior density *p*(*X*_*t*_|*y*_1 : *t*_) ≈ *N*(*m*_*t*_, Σ_*t*_). The lagged forward pass is then obtained from the application of a linear marginalization operator *M*, as follows:

A10 $$\begin{array}{c} p\left(x_{t-k}|y_{1:t}\right)\approx N\left(\mu_{t-k},S_{t-k}\right)\\ \mu_{t-k}=Mm_{t}\\ S_{t-k}=M\Sigma_{t}M^{T}\\ M≜\left[\begin{array}{cc} I_{n} & 0_{n\times n\left(k-1\right)} \end{array}\right] \end{array}$$

Eq. (A10) can be applied with any arbitrary lag *k*. This means that the entire time series can in principle be inverted in one step. However, there will be an optimal lag to consider for any state-space model, in terms of the balance between computational demand and precision of the estimators.

Note also that the Taylor expansion point can be chosen arbitrarily. If used in a recursive way, i.e.: $\Gamma_{t}\leftarrow {\left[\begin{array}{ccc} \mu_{t-k}{}^{T} & \cdots & \mu_{t}{}^{T} \end{array}\right]}^{T}$, then iterating the above lagged forward pass performs a Gauss–Newton ascent on the states' posterior probability, with the usual convergence properties. This is necessary in the context of the VBL update of the states' posterior. We refer to Daunizeau et al. (2009) for additional technical details.

## Footnotes

1 The second-order moments of generalized state-noise can be state-dependent (see, e.g., Friston et al., 2010). Furthermore, one can also decompose the inverse covariance matrix, known as the precision matrix, into a linear mixture of precision components.

2 This is strictly true because these systems are stable.
