# Supplementary material for: Stochastic dynamic causal modelling of fMRI data: Should we care about neural noise?
Source: Neuroimage. 2012 Aug 1;62(1):464–81. doi: 10.1016/j.neuroimage.2012.04.061 (PMC3778887; doi:10.1016/j.neuroimage.2012.04.061)
Supplement: Supplementary file 1 — Supplementary materials. [file mmc1.pdf]

Stochastic Dynamic Causal Modelling of fMRI data:

Should we care about neural noise?

SUPPLEMENTARY MATERIAL

J. Daunizeau[1,2], K.E. Stephan[1,2,3], K.J. Friston[1]

[1] Wellcome Trust Centre for Neuroimaging, University College of London, United Kingdom

[2] Laboratory for Social and Neural Systems Research, Dept. of Economics, University of Zurich, Switzerland

[3] Translational Neuromodeling Unit (TNU), Institute of Biomedical Engineering, University of Zurich & ETH Zurich, Switzerland

This note reports all the details of statistical analysis conducted on the Monte-Carlo simulations series of the results section of the main manuscript.

Monte-Carlo simulations: assessing estimation accuracy

As stated in the main text, this simulation series was designed to test whether one can improve DCM's unknown variables estimation by including stochastic state noise in the generative model. The following five experimental factors were systematically varied:

- *SNR*: The fMRI data signal-to-noise ratio
- Δu : The presence of neural fluctuations or neural noise
- *NL*: The presence of nonlinearities in the neural evolution function
- *DCM type*: Stochastic or deterministic DCM, i.e. the generative model assumed or not the presence of state noise.
- $\hat{u}$ *basis*: The use of non-specific input basis function sets $\hat{u}$ in the neural evolution function. This factor had three levels: (1) no input basis function set, (2) Fourier basis function set and (3) radial basis function (RBF) set.

The first three factors control the way the fMRI data were simulated. In contradistinction, the last two determine the generative model that was used for the ensuing DCM inversion. For each cell of this 2x2x2x2x3 factorial design, we measured both the estimation accuracy (MSE) and the confidence bias (MCB) on (i) network connectivity parameters, (ii) region-dependent inputs and (iii) neural states dynamics. We report below the details of the statistical analysis.

We first verified by an F-test (Table SP-1) that the five factors above captured variations in both MSE and MCB for all model's variables (inputs, states and evolution parameters).

	input	neural states	evolution parameters
MSE	F=7.8, $p<10^{-5}$	F=11.7, $p<10^{-5}$	F=15.6, $p<10^{-5}$
MCB	F=5.3, $p<10^{-5}$	F=23.5, $p<10^{-5}$	F=16.1, $p<10^{-5}$

Table SP-1. Omnibus F-test for ANOVA onto all dependent variables.

Starting with the results on neural states estimation accuracy, the 5-way interaction *SNR* x *NL* x Δu x *DCM type* x $\hat{u}$ *basis* is statistically significant for MSE (F=5.01, p=0.007) and the 4-way interaction *NL* x Δu x *DCM type* x $\hat{u}$ *basis* is statistically significant for MCB (F=4.3, p=0.013). This means that, strictly speaking, the effect of each of these factors depends on the level of (all) other ones (except *SNR* for MCB). The main effects are thus statistically meaningless (even though they confirm intuition; e.g., *SNR*, *NL* and Δu decrease estimation performance on average). First, note that for both MSE and MCB (Figure 5 and 8), *SNR*, Δu , *DCM type* and $\hat{u}$ *basis* only make a significant difference for nonlinear DCMs, i.e. no factor levels comparison passes the 5% significance threshold without nonlinearities (every other factor level being fixed). We thus inspect the results in the nonlinear case.

First, we report the effect of the input basis function set. For stochastic DCMs, the average effect of using either a Fourier or RBF input basis set either has a detrimental effect (on MSE, when *SNR* is low and without input perturbations, F=8.73, p=0.003), or has no significant effect (all other cases). This means that augmenting sDCM with input basis sets induces non-identifiability issues that arise from the redundancy in the neural noise representation. However, this does not change the quality of the credible intervals. (We will

come back to this when inspecting the estimation of evolution parameters, but will focus on stochastic DCM without input basis set in the following.)

The situation is different for deterministic DCM, which significantly benefits from the input basis set but only when there are input perturbations (F=65.2, $p<10^{-5}$ for MSE and F=78.1, $p<10^{-5}$ for MCB); this is irrespective of the SNR level. We will thus consider all variants of deterministic DCM in the following comparison with stochastic DCM.

For high *SNR*, sDCM performs significantly better than dDCM without input basis function set (only in the presence of neural noise, F = 42.6, $p<10^{-5}$ for MSE and F=46.4, $p<10^{-5}$ for MCB) but this difference disappears when dDCM is augmented with input basis function sets (F=0.8, p=0.37 for MSE and F=1.09, p=0.29 for MSB). Surprisingly for low *SNR*, there is a dissociation of MSE and MCB scores:

- In terms of MSE, there is no significant difference between sDCM and dDCM without input basis function set (even in the presence of neural noise: F=0.42, p=0.51). However, augmented dDCM performs significantly better than sDCM in the presence of neural noise (F=21.3, $p<10^{-5}$).
- In terms of MCB, sDCM performs significantly better than dDCM without input basis function set, irrespective of the actual presence of neural noise (F=24.9, $p<10^{-5}$), but this difference disappears when dDCM is augmented with input basis function sets (F=0.40, p=0.52).

Taken together, this means that accounting explicitly for the presence of neural noise (either using stochastic DCM or deterministic DCM augmented with input basis function sets) is critical for the quality of states estimation in the presence of nonlinearities. This conclusion is strengthened by looking at the following Δu x *DCM type* interactions:

At low *SNR* this interaction is significant for dDCM without input basis function set (F =33.0, $p<10^{-5}$ for MSE and F=18.4, $p<10^{-4}$ for MCB). This is because neural noise has an effect on dDCM (F=61.4, $p<10^{-5}$ for MSE and F=20.6, $p<10^{-5}$ for MCB) but not on sDCM (F=0.05,

p=0.82 for MSE and F=1.76, p=0.19 for MCB). However, this interaction disappears for dDCM augmented with input basis function sets (F=0.002, p=0.96 for MSE and F=0.16, p=0.68 for MCB). Indeed, neural noise has no significant effect on sDCM or augmented dDCM.

For low *SNR*, we observe again a dissociation between MSE and MCB scores:

- MSE: the above interaction is not significant for dDCM without input basis function set (F=0.04, p=0.84), but becomes significant for dDCM augmented with input basis function sets (F=10.6, p=0.001). Indeed, neural noise significantly deteriorates estimation accuracy for sDCM (F=28.0, $p<10^{-5}$) and dDCM without input basis set (F=35.7, $p<10^{-5}$), but it does not for dDCM with input basis set (F=3.79, p=0.052).
- MCB: the above interaction is significant for dDCM without input basis function set (F=6.66, p=0.01), but not for dDCM with input basis set (F=1.52, p=0.21). Indeed, neural noise has no effect on either sDCM (F=0.05, p=0.81) or dDCM with input basis function set (F=3.42, p=0.06), but does significantly aggravates the overconfidence of dDCM without input basis set (F=16.3, $p<10^{-4}$).

Turning now to our results on input estimation accuracy, we found that most of the high-order interactions are significant, e.g., the 4-way interaction *NL* x Δu x *DCM type* x $\hat{u}$ *basis* for MSE (F=3.88, p=0.02) and the 5-way interaction *SNR* x *NL* x Δu x *DCM type* x $\hat{u}$ *basis* for MCB (F=4.66, p=0.009).

To begin with, the effect of augmenting the model with input basis function sets was unexpected. In brief, it is either MSE-detrimental to both sDCM (F=41.9, $p<10^{-5}$, in the nonlinear case but irrespective of *SNR* or Δu) and dDCM (F=10.6, p=0.001, in the nonlinear case and with neural noise but irrespective of *SNR*), or has no effect (all other cases). We will discuss this later, but this justifies the fact that we will focus on the stochastic-deterministic comparison, without input basis function set in the following.

For MSE (Figure 8), there is no significant difference between sDCM and dDCM, irrespective of the levels of all the other factors. The situation is quite different for MCB (Figure 11). First, in the absence of nonlinearities, dDCM is significantly more overconfident than sDCM (F=8.81, p=0.003, irrespective of *SNR* or Δu). Second, in the presence of nonlinearities, the three-way *SNR* x *DCM type* x Δu interaction is significant (F=5.78, p=0.01). Closer inspection shows that both two-way *DCM type* x Δu interactions for high *SNR* (F=7.7, p=0.005) and low *SNR* (F=23.3, $p<10^{-5}$) are significant. In brief: (i) all one-to-one s/d comparison show that dDCM is significantly more overconfident than sDCM and (ii) this difference is significantly amplified when *SNR* decreases and when neural noise is present.

Taken together, this means that, in terms of the quality of neural noise estimation, stochastic DCM performs much better than any variant of deterministic DCM.

Finally, we report the results on the evolution parameters estimation accuracy (Figures 7 and 10). Concerning the effect of input basis function set, the following 3-way interactions were significant: *NL* x *DCM type* x $\hat{u}$ *basis* (F=7.84, $p<10^{-3}$ for MSE and F=11.2, $p<10^{-4}$ for MCB) and *NL* x Δu x $\hat{u}$ *basis* (and F=22.8, $p<10^{-5}$ for MSE and F=22.7, $p<10^{-5}$ for MCB). We thus have to analyze its effect on sDCM and dDCM separately. In brief:

- sDCM: the average effect of using either a Fourier or RBF input basis set always has a detrimental effect on both MSE and MCB, except when there are input perturbations and *SNR* is high (no effect).
- dDCM: surprisingly, augmenting the model with either a Fourier or RBF input basis set has no significant effect on MSE or MCB, irrespective of any other factor.

In the following, we will thus focus on DCM without input basis set.

None of the high-order interactions are significant on MSE, except for the two-way interaction Δu x *DCM type* (F=7.49, p=0.06). This is because estimation error significantly increases in the presence of neural noise for dDCM (F=7.82, p=0.005) but not for sDCM (F=0.44, p=0.51). The absence of interactions involving *SNR* and nonlinearities means we can test for their main effect on estimation error, but neither is significant (F=2.46, p=0.12 for *NL* and F=0.40, p=0.53 for *SNR*).

The situation is similar for MCB: none of the higher-order interactions are significant, except for the two-way interaction *NL* x *DCM type* (F=7.09, p=0.07). This is because sDCM is significantly more overconfident than dDCM in the absence of nonlinearities (F=6.21, p=0.013), but this difference disappears in the presence of nonlinearities (F=1.72, p=0.19). Note that the main effects of the other factors are not significant (F=0.36, p=0.55 for *SNR* and F=1.42, p=0.23 for Δu).

Monte-Carlo simulations: assessing model comparison

As stated in the main text, this simulation series was designed to ask whether the presence of state noise can help discriminating between two network structures A and B. The following three experimental factors were systematically varied:

- *network*: fMRI data were simulated either under model A (mediated influence) or under model B (direct and mediated influence).
- Δu : the simulated system was either driven (unilaterally) by an exogenous input u or by the same input plus some neural fluctuation Δu .

- *DCM type*: the generative model assumed presence or absence of state noise ϖ (i.e., stochastic or deterministic DCM).

The first two factors control the way the fMRI data were simulated. In contradistinction, the last factor determines the generative model that was used for the ensuing DCM inversion. For each cell of this 2x2x2 factorial design, we measured the relative evidence in favour of network A (the log- Bayes factor LBF_{AB}). We report below the details of the statistical analysis.

An initial F-test confirmed (F=2.59, p=0.01) that the three factors (*network*, Δu and *DCM type*) captured the variations of LBF_{AB}. We then tested for the sign of LBF_{AB} as a function of whether the simulated data were generated under model A or model B. It turned out that in the presence of neural noise only sDCM could distinguish the two networks, i.e. LBF_{AB} was significantly greater than zero when *network*=A (t=1.71, p=0.04) and significantly smaller than zero when *network*=B (t=3.57, p=0.001).

The only significant interaction was the 2-way interaction *network* x *DCM type* (F=12.33, p=5.8 10^{-4}). This means that the result of Bayesian model comparison depended on the type of DCM (sDCM or dDCM). When conditioning on Δu, the interaction *network* x *DCM type* was not significant in the absence of state noise (F=3.06, p=0.08), but was significant in the presence of state noise (F=11.02, p=0.001). Similarly, state noise affected the difference of LBF_{AB} between network types:

- for absent state noise, neither sDCM (F=3.34, p=0.07) nor dDCM (F=0.04, p=0.83) showed a significant difference;
- in the presence of state noise, this difference became significant for sDCM (F=14.28, p=2.3 10^{-4}) but nor for dDCM (F=0.14, p=0.71).
